# Breeding progress of disease resistance and impact of disease severity under natural infections in winter wheat variety trials

**DOI:** 10.1007/s00122-020-03728-4

**Published:** 2021-03-13

**Authors:** F. Laidig, T. Feike, S. Hadasch, D. Rentel, B. Klocke, T. Miedaner, H. P. Piepho

**Affiliations:** 1 Institute of Crop Science, Biostatistics Unit, University of Hohenheim, Fruwirthstrasse 23, 70599 Stuttgart, Germany; 2 Institute for Strategies and Technology Assessment, Julius Kühn-Institut (JKI), Federal Research Centre for Cultivated Plants, Stahnsdorfer Damm 81, 14532 Kleinmachnow, Germany; 3 Bundessortenamt, Osterfelddamm 60, 30627 Hannover, Germany; 4 University of Hohenheim, State Plant Breeding Institute, Fruwirthstrasse 21, 70599 Stuttgart, Germany

## Abstract

**Key message:**

Breeding progress of resistance to fungal wheat diseases and impact of disease severity on yield reduction in long-term variety trials under natural infection were estimated by mixed linear regression models.

**Abstract:**

This study aimed at quantifying breeding progress achieved in resistance breeding towards varieties with higher yield and lower susceptibility for 6 major diseases, as well as estimating decreasing yields and increasing disease susceptibility of varieties due to ageing effects during the period 1983–2019. A further aim was the prediction of disease-related yield reductions during 2005–2019 by mixed linear regression models using disease severity scores as covariates. For yield and all diseases, overall progress of the fully treated intensity (I2) was considerably higher than for the intensity without fungicides and growth regulators (I1). The disease severity level was considerably reduced during the study period for mildew (MLD), tan spot (DTR) and Septoria nodorum blotch (ear) (SNB) and to a lesser extent for brown (leaf) rust (BNR) and Septoria tritici blotch (STB), however, not for yellow/stripe rust (YLR). Ageing effects increased susceptibility of varieties strongly for BNR and MLD, but were comparatively weak for SNB and DTR. Considerable yield reductions under high disease severity were predicted for STB (−6.6%), BNR (−6.5%) and yellow rust (YLR, −5.8%), but lower reductions for the other diseases. The reduction for resistant vs. highly susceptible varieties under high severity conditions was about halved for BNR and YLR, providing evidence of resistance breeding progress. The empirical evidence on the functional relations between disease severity, variety susceptibility and yield reductions based on a large-scale multiple-disease field trial data set in German winter wheat is an important contribution to the ongoing discussion on fungicide use and its environmental impact.

**Electronic supplementary material:**

The online version of this article (10.1007/s00122-020-03728-4) contains supplementary material, which is available to authorized users.

## Introduction

Wheat is one of the world’s most important staple foods and is susceptible to several important plant diseases (Savary et al. 2019). In the European Union, Germany is the second largest wheat-growing country after France (Fones and Gurr 2015) with an average acreage of about 3.1 million ha (mean 2015–2019), which corresponds to 27% of the country’s total arable land (Stat J 2019). The on-farm yield in Germany rose considerably from about 55 dt ha^−1^ in 1983 to more than 76 dt ha^−1^ to date (mean 2015–2019) (Stat J 2019). This increase was achieved by higher-input cropping systems based on improved crop management, improved fertilizer application, more effective crop protection measures and new improved varieties with higher yield potential and higher resistance to diseases. However, yield losses due to fungal diseases are hampering progress for higher on-farm yields in winter wheat.

Numerous studies on yield loss in winter wheat due to fungal diseases have been published (e.g. Savary et al. 2006; Zhang et al. 2006; Loyce et al. 2008; Fones and Gurr 2015; Jevtic et al. (2017). Oerke and Dehne (2004) report a global yield loss potential due to fungal diseases of 16% for wheat. Savary et al. (2019) report an expert-based assessment of yield losses of 25% due to pathogens and pests in winter wheat for the wheat-growing areas of North-West Europe, including Germany. The loss was nearly completely attributed to fungal disease damage, where yellow rust (syn. stripe rust) and Septoria tritici blotch were indicated as the diseases causing the highest yield losses.

Fones and Gurr (2015) considered Septoria tritici blotch, caused by the fungus *Zymoseptoria tritici*, as the most problematic foliar disease of wheat among the various pathogens of the humid climatic region that includes Northern France, Germany and the UK. They state that this fungus shows a degree of evolutionary “plasticity” which may allow it to keep pace with innovations in disease control with relative ease. This persistent pathogen is considered responsible for approximately 70% of annual fungicide usage in the EU wheat production (Fones and Gurr 2015).

While global demand for agricultural products is continuously increasing, the public debate on the use of pesticides and the negative environmental impact of intensive crop production is increasing. It is hence a persistent challenge to realize higher yields with less environmental impact, i.e. a sustainable intensification of crop production. It is an important goal of agricultural policies in the European Union, including Germany, to reduce the surplus of nitrogen fertilizer and application of pesticides to mitigate the negative impact on the environment and improve the sustainability of plant production (BMEL 2019; EU 2019). The European Commission aims to reduce the overall use and risk of chemical pesticides by 50% and the use of more hazardous pesticides by 2030 (EU 2020).

Against this background, new improved varieties are a key factor to generate higher on-farm yields. Considerable breeding efforts have been undertaken not only to raise potential yield and baking quality (Laidig et al. 2017) of new varieties, but also to improve disease resistance. Most studies on disease susceptibilities and yield loss were done with limited experimental data. Loyce et al. (2008) investigated the interaction of cultivar and crop management on winter wheat diseases in trials conducted in 3 years at 18 locations with 20 varieties. Breeding progress in winter wheat variety trials for disease resistance under natural infection was reported for example by Ahlemeyer and Friedt (2011) and Voss-Fels et al. (2019) using historic varieties grown in 3 years at 10 environments and in two years at twelve environments, respectively. Zetzsche et al. (2019) evaluated breeding progress of disease resistance for historic European winter wheat varieties, which were artificially inoculated in greenhouse seedling tests. Zhang et al. (2006, 2007) investigated variety susceptibilities and yield losses in multiple disease systems of French winter wheat variety trials grown over 13 years (1990–2002) in up to 99 trials per year under artificial inoculation. To the best of our knowledge no study exists that investigated breeding progress with regard to natural disease severity and yield loss over that many years, sites and genotypes as included in this investigation based on the archives of testing authorities. The extent of those data makes them very useful in retrospective studies (e.g. Mackay et al. 2011) aimed at improving the understanding of the interrelation of chemical plant protection, resistance breeding and yield loss, especially with respect to the intended political measures to reduce the use of pesticides. The focus on natural infections here represents the situation the farmer is facing daily.

In our study, we analyse a dataset based on official German variety registration trials. The analysis aims, first, to quantify the long-term change of yield and disease severity of 6 major fungal diseases of winter wheat, second, to evaluate the effect of variety age on yield potential and disease susceptibility of varieties and, third, to predict yield reduction under natural multiple disease severity conditions relative to fungicide- and growth regulator-controlled yields.

## Materials and methods

### Trials for testing a variety’s value for cultivation and use

In Germany, new candidate varieties are evaluated for their value for cultivation and use by the Federal Plant Variety Office (Bundessortenamt, BSA). Only if a new variety offers significant advantages over existing varieties, it is approved for release to the market. Major evaluation criteria for variety registration include grain yield quantity and quality parameters. Additionally, it considers the susceptibility of winter wheat to 8 fungal diseases: Eyespot (*Oculimacula yallundae*, syn. *Pseudocercosporella herpotrichoides*), powdery mildew (*Blumeria graminis*), yellow (stripe) rust (*Puccinia striiformis*), brown (leaf) rust (*Puccinia triticina*), Septoria nodorum blotch (*Parastagonospora nodorum*), Septoria tritici blotch (*Zymoseptoria tritici*), Fusarium head blight (*Gibberella zeae*, syn. *Fusarium graminearum*) and tan spot (*Drechslera tritici-repentis*). The diseases are of different importance, some occurring frequently, others only occasionally, but they are all considered in the evaluation of a new variety. Fusarium head blight and eyespot are not included in this study, because they are evaluated based on data observed in separate trials under artificial infection.

**Fig. 1:**
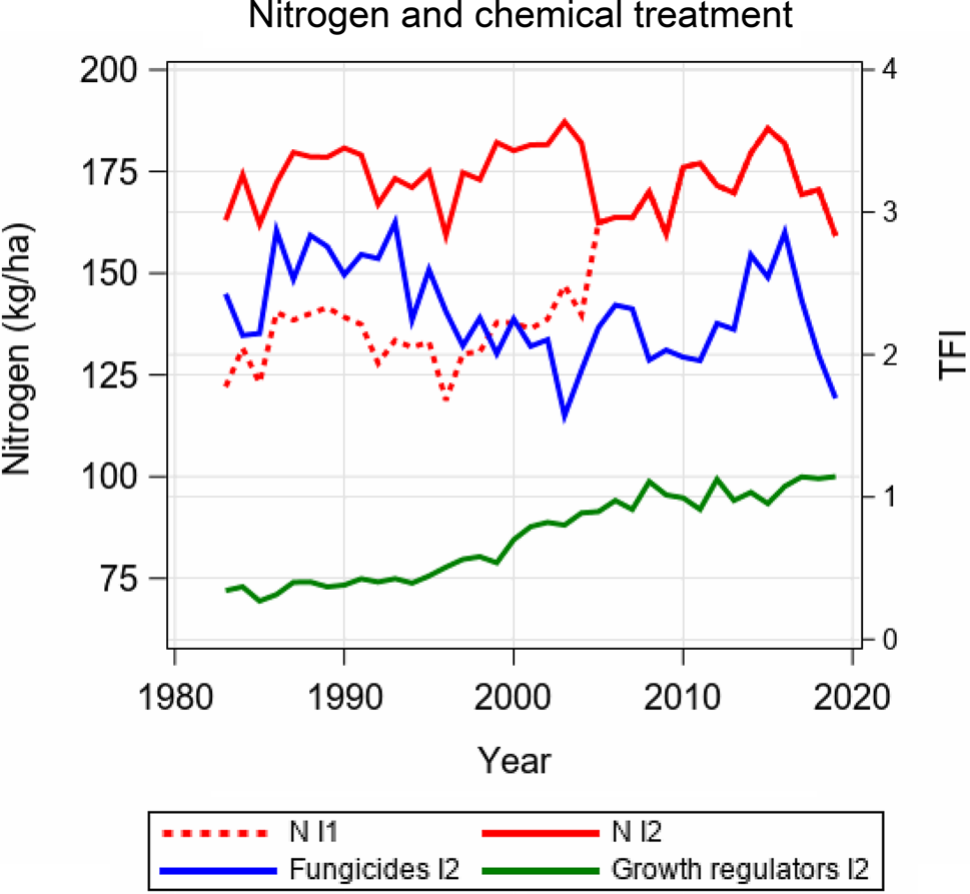
Applied average nitrogen rate for intensity I1 and I2 and treatment frequency index (TFI) for fungicides and growth regulators applied to I2

The testing period for newly submitted varieties in registration trials in Germany is 3 years. During the studied period from 1983 to 2019, every year about 100 candidate varieties entered testing; thereof, on average about 15 varieties were registered every year for their “value for cultivation and use (VCU)”, i.e. accepted for release as new varieties on the market. Varieties were grown in trial series at up to 30 locations per year with 2–3 replications in each trial. Single plot size was about 12 m^2^. The trials were evenly distributed across the typical winter wheat-growing regions in Germany. Within one year three trial series were conducted. Series 1, 2 and 3 included varieties being in the first, second and third testing year, respectively. Additionally, at least three reference varieties were included in each trial series, which were identical across series and over several years. A reference variety was grown in the trials for about seven years on average. Well-established varieties were chosen as references representing the actual state of breeding progress. References were updated on a regular basis, ensuring at least partial overlap of sets of references used in successive years. Before 1990, only data from West German locations are available. Trials were conducted with up to 4 intensities of different application rates for nitrogen, fungicides and growth regulators, where intensity 1 (hereinafter referred to as I1) received generally no fungicides or growth regulators and less nitrogen. From 1991 onwards, varieties were tested with only 2 intensities. From 2005 onwards, the nitrogen application rates were standardized and both intensities received the same nitrogen rates (see Fig. 1). In order to be able to consider 2 intensities in every year over the entire time series, we averaged data for intensity 2, intensity 3 and intensity 4 (hereafter referred to as I2, I3 and I4, respectively) for the period 1983–1990 and used this average as I2. Herbicides and insecticides were applied for all intensities at the same level. Following a standard procedure, in I2 at each site and year, fungicides were applied at the same dates and dosages over all varieties independently of the actual variety-specific disease intensity and resistance level. Besides grain yield (YLD), data for 6 major fungal diseases were included in this study: powdery mildew (MLD), brown rust (BNR), Septoria tritici blotch (STB), Septoria nodorum blotch (SNB), yellow rust (YLR) and tan spot (DTR) (see Table 1). In the early years of the data used in this study, both Septoria diseases on the leaf were estimated in the trials as one rating, because the visual symptoms were hard to differentiate. Later on, STB became predominant, while SNB lost its importance in leaf and ear infections. Data on tan spot severity were only available from the year 1996 onwards; consequently, all related analysis and results are based on data 1996–2019.

**Table 1:** Winter wheat fungal diseases investigated in the present study, their causal agents, abbreviations used in this study and respective EPPO codes (EPPO, 2019); alternative common names are given in parentheses

Abbreviation	EPPO^a^ code	Fungal disease	Causal agent
MLD	ERYSGT	Powdery mildew	*Blumeria graminis* f. sp. *tritici* (formerly, *Erysiphe graminis* f. sp. tritici)
BNR	PUCCRT	Brown (leaf) rust	*Puccinia triticina* (*Puccinia recondita f.sp. tritici*)
STB	SEPTTR	Septoria tritici blotch (Septoria leaf blotch)	*Zymoseptoria tritici* (*Septoria tritici*)
DTR	PYRNTR	Tan spot (yellow leaf blotch)	*Pyrenophora tritici-repentis* (*Drechslera tritici-repentis*)
YLR	PUCCST	Yellow rust (Stripe rust)	*Puccinia striiformis* f.sp. *tritici*
SNB	LEPTNO	Septoria nodorum blotch (Glume blotch)	*Parastagonospora nodorum* (*Septoria nodorum*) (*Phaeosphaeria nodorum*)

^a^European and Mediterranean Plant Protection Organization

### Visual assessment of disease severity

Disease severity was assessed visually by crop experts in the field according to the guidelines of the Federal Plant Variety Office (Bundessortenamt 2000) at 2–3 different growth stages for each disease using the BBCH-code (Hack et al. 1992). It should be stated clearly that in the VCU trials of this study, plants were not inoculated with diseases, but disease severity was caused by natural field infection. If disease severity was assessed up to growth stage BBCH 32 (stem elongation, 2 nodes detectable), then it was scored at the whole-plant level. After growth stage BBCH 32, severity was assessed at the 2 adjacent leaves showing the highest severity. The 2 leaves were determined specifically for each plot and assessment, and the same leaves were used for all plants in this plot. Then the whole plot severity is assessed as the average of the infected area over all plants. The degree of the average disease severity of a plot was determined by a 1–9 scale, as shown in Table 2.

**Table 2:** Disease severity scores as a function of diseased leaf/spikelet area (Bundessortenamt 2000)

Disease severity		
Score	State	Diseased leaf/ spikelet area (%)
1	Missing	0
2	Very low to low	> 0–2
3	Low	> 2–5
4	Low to medium	> 5–8
5	Medium	> 8–14
6	Medium to high	> 14–22
7	High	> 22–37
8	High to very high	> 37–61
9	Very high	> 61–100

The transformation of the percentage of disease-infected leaf area of a plot into a 1–9 scale of scores corresponds approximately to a logarithmic transformation (Bundessortenamt 2000). The recorded score represents the average disease severity of the plot. Two border rows at each plot side and one meter of each plot head were not included in the plot assessment area. The observations were carried out at each replication of I1–I4 and for each variety. For the variety registration process, only observations of that growth stage were used, which showed the most clearly visible severity differentiation (i.e. not necessarily the maximum) among varieties. According to Zadoks and Schein (1979, p. 64), this method can be considered as the “critical time method”. Observed disease severity scores at this growth stage were averaged over replications. Only these average values were available for the present study’s analyses.

Throughout this paper we use the following terms: (1) “disease severity” to describe each individual variety’s actual visually observed diseased leaf or spikelet area, expressed in the above-described “scores” (Table 2), (2) “trial disease severity (TSv)” to describe the average disease severity within a trial, calculated as the mean severity score over all varieties in the specific trial, (3) “variety disease susceptibility (VSc)” to refer to the average visible disease severity of a variety, calculated as the mean severity score across all diseased trials in which this specific variety was grown. Disease susceptibility of a variety may also be considered as the inverse of its “disease resistance”. A trial is considered as non-diseased with respect to a specific disease, if no disease symptoms for the specific disease were visible for all varieties in this trial, or, if only a few varieties showed a very low severity. (4) The 1–9 scale with scale unit “score” is used to express results for diseases without always indicating the underlying 1–9 scale or specifying the measurement unit.

### Data

#### Yield and diseases

Data from German official variety trials were analysed over the period of 37 years from 1983 to 2019 originating from 1755 individual trials. The data set comprised 789 genotypes having trial results of at least three years. Thereof, 387 genotypes were finally released. Data from varieties for which testing was not continued after the first or second year were not included in this study. The reason for including 392 varieties which were not released after the third testing year, was to achieve a better representation of the trial conditions and use of a more solid database. On average, 47 trials were available each year and 27 varieties grown in each trial. There were 56 reference varieties, represented by more than 3 trial years. In total, 47,318 observations were analysed. The oldest reference variety included in our data set was first tested in 1963, i.e. the time of a variety’s first year in trial spanned a period from 1963 to 2017, covering 55 years of breeding progress. The data set was highly non-orthogonal with respect to variety-year combinations, whereas the variety-location combinations were orthogonal within year and trial series, i.e. all varieties were grown together at all locations within the same year and trial series. Only about 1.4% of the possible variety-location-year combinations were present.

In order to avoid biased results, we checked our data thoroughly for consistency in structure over time before carrying out the analysis by, e.g. investigating the number of locations and their distribution across Germany within each year, and removing durum winter wheat varieties. The data were further checked for recording errors and outliers by calculating standardized residuals based on model Eq. (1a) and (1b). Observations with standardized residuals greater than ± 5.0 were excluded from further analysis. In total, 0.13% of the observations exceeded the threshold and were therefore dropped.

#### Fertilizer, fungicides and growth regulator

The fertilizer, fungicide and growth regulator rates were recorded in detail for each individual trial and intensity. Nitrogen application rates were accumulated as total kg N ha^−1^. The preceding crops’ residual nitrogen supply was considered based on DUEV (2017, Fig. 7) and added to the applied mineral N rate. The nitrogen equivalent of sporadically applied organic fertilizer was considered and added according to the applied mineral N rate. Unfortunately, no data on plant-available mineralized nitrogen in the soil (Nmin) before the vegetation period were available. The total fungicide and growth regulator application rates were standardized using the treatment frequency index (TFI) as described by Roßberg (2006). The TFI is a commonly applied index for assessing plant protection intensities (e.g. Schwarz et al. 2018; Strehlow et al. 2020). The TFI describes the amount of plant protection products applied to a specific land unit relative to the application amount recommended by the approval authority for each individual plant protection product for the specific crop. A TFI of 1 may derive from the application of a single plant protection product in recommended full dose, but may also derive from the application of 2 plant protection products, each applied at half the recommended dose. The TFI is often derived separately for fungicides, herbicides, insecticides and growth regulators. Accordingly, we derived separate TFIs for fungicide and growth regulator applications in our study. The average rate of applied nitrogen, fungicides and growth regulators is shown in Fig. 1.

### Statistical analysis

#### General remarks

Diseases were assessed on an ordinal 1–9 scale, but we analysed them as being on a quantitative scale. This is standard procedure in the analysis of variety trials, and in fact, decisions for registration of new varieties are based on such analyses. Whereas the data cannot meet the usual assumptions of normality and homogeneity of variance, residual analysis revealed no gross departures (we refer to this later in the Discussion under Statistical aspects), so we analysed these data as if they were on a quantitative scale. Further, we did not dissect genetic and non-genetic components of trends by expanding the basic model (Eq. 1a) using fixed regression terms for both components (Piepho et al. 2014), because we encountered some problems leading to unrealistic results, which we will pick up later in the section Transformation vs. no transformation of visual observations on the 1–9 scale.

#### Basic model

For a given intensity we used the standard three-way model with factors genotype, location and year given by Laidig et al. (2008).

1a $$y_{ijk} = \mu + G_{i} + L_{j} + Y_{k} + \left( {LY} \right)_{jk} + \left( {GL} \right)_{ij} + \left( {GY} \right)_{ik} + \left( {GLY} \right)_{ijk}$$

where *y*_*ijk*_ is the mean yield of the *i*th genotype in the *j*th location and *k*th year, *μ* is the overall mean, *G*_*i*_ is the main effect of the *i*th genotype, *L*_*j*_ is the main effect of the *j*th location, *Y*_*k*_ is the main effect of the *k*th year, (*LY*)_*jk*_ is the *jk*th location × year interaction effect, (*GL*)_*ij*_ is the *ij*th genotype × location interaction effect, (*GY*)_*ik*_ is the *ik*th genotype × year interaction effect, and $\left( {GLY} \right)_{ijk}$ is a residual comprising both genotype × location × year interaction and the error of a mean arising from sampling the replications. All effects except *μ*, and *Y*_*k*_ are assumed to be random and independent with constant variance for each effect.

#### Basic model for yield

In 2005 the rate of nitrogen fertilization was changed in such a way that plots of I1 and I2 received the same N-rate per ha until 2019 (Fig. 1). Before 2005 the nitrogen level in I1 was lower than in I2. We considered this change by adding a fixed categorical effect *P*_*l*_ in Eq. (1a) with 2 levels, replacing *μ* by *P*_1_ for years 1983–2004 and by *P*_2_ for years 2005–2019.

The extended model is given by

1b $$y_{ijkl} = P_{l} + G_{i} + L_{j} + Y_{k} + \left( {LY} \right)_{jk} + \left( {GL} \right)_{ij} + \left( {GY} \right)_{ik} + \left( {GLY} \right)_{ijk}$$

#### Model for overall yield trend

An overall trend was modelled considering the genotypes as nested within years (Laidig et al. 2014). Thus, compared with model Eq. (1a) and (1b), we dropped effects involving genotypes that are not nested within years, i.e. the effects $G_{i}$ and (*GL*)_*ij*_. The reduced model for yield is then given by

2a $$y_{ijkl} = P_{l} + L_{j} + Y_{k} + \left( {{LY}} \right)_{jk} + \left( {GY} \right)_{ik} + \left( {GLY} \right)_{ijk}$$

#### Model for overall disease trend

For diseases we did not consider the change in nitrogen treatment, because we found that the categorical effect *P*_*l*_ was not significant (*p* > 0.05) for all diseases (results not shown), indicating that this change had no major influence on disease response in both periods. The reduced model for diseases is given by

2b $$ y_{ijk} = \mu + L_{j} + Y_{k} + \left( {LY} \right)_{jk} + \left( {GY} \right)_{ik} + \left( {GLY} \right)_{ijk}$$

We expressed *Y*_*k*_ in Eqs. (2a and 2b) as a quadratic function of time by

2c $$Y_{k} = \alpha t_{k} + \beta t_{k}^{2} + U_{k}$$

where $\alpha$ is a fixed linear and $\beta$ a fixed quadratic regression coefficient for overall trend, *t*_*k*_ is the continuous covariate for the calendar year, and *U*_*k*_ is a random residual following a normal distribution with zero mean and variance $\sigma_{U}^{2}$.

#### Model for trend of variety means

The trend of variety means was based on a quadratic model given by

3 $$y_{i} = \mu + \alpha r_{i} + \beta r_{i}^{2} + C_{m\left( i \right)} + V_{i}$$

where $y_{i}$ is the mean severity score of genotype *i* averaged over all trials in which the *i*th genotype was present (VSc); the covariate *r*_*i*_ is the first trial year of the *i*th genotype. There are *m* groups of genotypes with the same first testing year *r*_*i*_, represented by categorical variable *C*_*m(i)*_, where the *i*th genotype was assigned to the *m*th group. *C*_*m(i)*_ is a random deviation of the *m*th group from the quadratic regression line with variance $\sigma_{C}^{2} ,$ and *V*_*i*_ a random deviation of the *i*th genotype from group *C*_*m(i)*_ with constant variance $\sigma_{V}^{2}$. The mean $y_{i}$ is a measure of the susceptibility of variety *i*, denoted by VSc.

#### Model for variety ageing

We compared the effect of variety age on yield and diseases by using pairwise differences of I2 and I1. Then $d_{ijkl} = \left( {y_{ijkl}^{2} - y_{ijkl}^{1} } \right)$ represents the difference of yield I2 and I1 and $d_{ijk} = \left( {y_{ijk}^{2} - y_{ijk}^{1} } \right)$ for diseases of the *i*th variety at the *j*th location and in the *k*th year, and the superscripts 1 and 2 for *y* denote the yield observed in I1 and I2, respectively. The fixed part of the model for overall trends (Eqs. 2a, b and c) has the quadratic regression equation

4a $$\eta_{kl} = P_{l} + \alpha t_{k} + \beta t_{k}^{2} \quad {\text{for yield and}}$$

4b $$\eta_{k} = \mu + \alpha t_{k} + \beta t_{k}^{2} \quad {\text{for diseases}}.$$

If we now assume that a quadratic age-dependent trend exists, then Eq. (4a) extends to

5a $$\eta_{ikl} = P_{l} + \alpha t_{k} + \beta t_{k}^{2} + \delta_{1} a_{ik} + \delta_{2} a_{ik}^{2}$$

and Eq. (4b) to

5b $$\eta_{ik} = \mu + \alpha t_{k} + \beta t_{k}^{2} + \delta_{1} a_{ik} + \delta_{2} a_{ik}^{2}$$

where $a_{ik} = r_{i} - t_{k}$ is the age of the *i*th variety at testing year *t*_*k*,_ and *δ*_1_ denotes the linear and *δ*_2_ the quadratic regression coefficient. We further assume that for I2 and I1 the overall trend in Eq. (5a and b) is identical, such that the fixed part of the difference I2–I1 of the regression models can be written as

6a $$\eta_{ikl}^{2} - \eta_{ikl }^{1} =\Delta P_{l} + \left( {\delta_{1}^{2} - \delta_{1}^{1} } \right)a_{ik} + \left( {\delta_{2}^{2} - \delta_{2}^{1} } \right)a_{ik}^{2} \:$$

for yield and for diseases,

6b $$\eta_{ik}^{2} - \eta_{ik}^{1} =\Delta \mu + \left( {\delta_{1}^{2} - \delta_{1}^{1} } \right)a_{ik} + \left( {\delta_{2}^{2} - \delta_{2}^{1} } \right)a_{ik}^{2}$$

where $\Delta P_{l} = P{}_{l}^{2} - P_{l}^{1}$, $\Delta \mu = \mu^{2} - \mu^{1}$. In Eq. (6a and b), superscripts 1 and 2 for *η, P* and *δ* now denote intensities I1 and I2, respectively.

If fungicide treatment fully compensates a variety’s loss of disease resistance in I2, then we can assume that the age effect in I2 is zero, meaning that $\delta_{1}^{2}$ = 0 and $\delta_{2}^{2}$ = 0 in Eqs. (6a and b). However, it should be emphasized that we do not assume that fungicide treatment fully controls disease, i.e. that disease severity is 1 on the 1–9 scale. We should also note that the age trend for yield will be positive because yield of varieties in I1 will decrease with increasing variety age *a*_*ik*_, where for diseases the susceptibility of varieties is likely to increase with increasing age, i.e. the susceptibility under I1 is increasing, meaning that we expect an age trend with negative sign.

When applying models Eq. (1a) and (1b) to treatment differences under the assumption that $\delta_{1}^{2} = 0$ and $\delta_{2}^{2} = 0$, i.e. that for I2 no age trend is present, then the trend of variety age can be incorporated into the genotype-year interaction term, which gives

6c $$\left( {GY} \right)_{ik}^{d} = \left( { - \delta_{1}^{1} } \right)a_{ik} + \left( { - \delta_{2}^{1} } \right)a_{ik}^{2} + \left( {DH} \right)_{ik}^{d}$$

where the superscript *d* denotes the difference between the random effects of I1 and I2 and $\left( {DH} \right)_{ik}^{d}$ is the deviation from the fixed part of Eq. (6c)

#### Extended model for impact of disease severity on yield (Model I)

It is of interest to quantify the effect of multiple natural disease severity on the relative yield of I1. We therefore expressed yield at I1 as percentage of yield at I2. To evaluate the effect of disease severity on yield I1, we extended model (1a) by considering all 6 disease severity scores as covariates using a second-order polynomial. For this analysis we used data only for growing years 2005–2016, first, to avoid potentially biased results due to different nitrogen levels in I1 and I2 before 2005, and second, to base the results on more recent and hence more relevant data. For this period, I1 was subject to exactly the same treatment except that no fungicides and growth regulators were applied. Therefore, the categorical effect P_*l*_ in Eq. (2a) was no longer necessary to model the different nitrogen application periods. To select the covariates that influence relative yield at I1, a forward selection procedure was carried out involving 2 steps. The basic model was Eq. (1a). We included the covariates for the quadratic overall trend (Eq. 2c) and for the 6 diseases. In the first stage, we selected the linear terms, in the second the quadratic ones. Covariates were added sequentially to the basic model, one at a time.

In the variable selection procedure, a coefficient of determination for mixed models (Piepho 2019) served to select covariates. This measure is equivalent to the adjusted coefficient of determination in a linear mixed model and is computed as given by

$$R^{2} = \frac{{\theta \left( {V_{0} } \right) - \theta \left( V \right)}}{{\theta \left( {V_{0} } \right)}}100\%$$

where $\theta \left( {V_{0} } \right) = trace\left( {V_{0} } \right)$ represents the trace of the variance–covariance matrix of the basic model and accordingly $\theta (V)$ is the trace of the model including covariates. $\theta \left( {V_{0} } \right)$ is the average marginal variance (AMV) for the baseline model (Piepho 2019). This measure was used to select both linear and quadratic covariates. By these 2 selection steps, the covariates that maximized $R_{ }^{2}$ were included in the final model (Description see Supplementary Material SM1). As inclusion of DTR did not improve the model fit; it was excluded.

The expected value of the final model is given by

7a $$ E\left( {z_{ijk} } \right) = \mu + \alpha_{1} {MLD}_{ijk} + \alpha_{2} {BNR}_{ijk} + \alpha_{3} {STB}_{ijk} + \alpha_{4} {SNB}_{ijk} + \alpha_{5} {YLR}_{ijk} + \beta_{1} {BNR}^{2}_{ijk} + \beta_{2} {STB}^{2}_{ijk}$$

where $z_{ijk} = 100 \times \left( {y^{1}_{ijk} /y^{2}_{ijk} } \right)$ for the *i*th variety in the *j*th year and the *k*th location, *MLD*, *BNR*, *STB*, *SNB* and *YLR* are the *ijk*th disease severity scores of the covariates, and *α*, *β* and *γ* are the regression coefficients of the linear, quadratic and covariates, respectively. Superscripts 1 and 2 for *y* denote intensities I1 and I2, respectively. We will refer to this model as prediction Model I.

#### Extended model for impact of trial severity and variety susceptibility on yield (Model II)

In this model, we predict the relative yield $z_{ijk}$ at I1 by the trial disease severity TSv and the variety disease susceptibility VSc. TSv is represented by the average severity score of all varieties grown in I1 in the same trial, where VSc is the individual variety’s average severity score in I1, observed over all trials in which the variety was grown. The aim of Model II is to demonstrate the interaction of TSv with the VSc. The covariates were selected using the analogous procedure as applied for Eq. (7a) and outlined in Supplementary Material SM1. The expected value of the final model is given by

7b $$ E\left( {z_{ijk} } \right) = \mu + \alpha_{1} mld_{i}^{V} + \alpha_{2} bnr_{i}^{V} + \alpha_{3} stb_{i}^{V} + \alpha_{4} snb_{i}^{V} + \alpha_{5} {{ylr}}_{i}^{V} + \beta_{1} {{mld}}_{i}^{V} \times {{mld}}_{jk}^{T} + \beta_{2} {{bnr}}_{i}^{V} \times {{bnr}}_{jk}^{T} + \beta_{3} {{stb}}_{i}^{V} \times {{stb}}_{jk}^{T} + \beta_{4} {{snb}}_{i}^{V} \times {{snb}}_{jk}^{T} + \beta_{5} {{ylr}}_{i}^{V} \times {{ylr}}_{jk}^{T} + \gamma_{1} {{stb}}_{i}^{V} \times ({{stb}}_{ik}^{T} )^{2}$$

where *mld*^*T*^, *bnr*^*T*^, *stb*^*T*^, *snb*^*T*^ and *ylr*^*T*^ denote the TSv, indicated by superscript *T*, covariates with the superscript *V* denote the Vsc, and *α*, *β* and *γ *are the corresponding regression coefficients. We will refer to this model as prediction Model II.

## Results

### Diseased trials

**Fig. 2:**
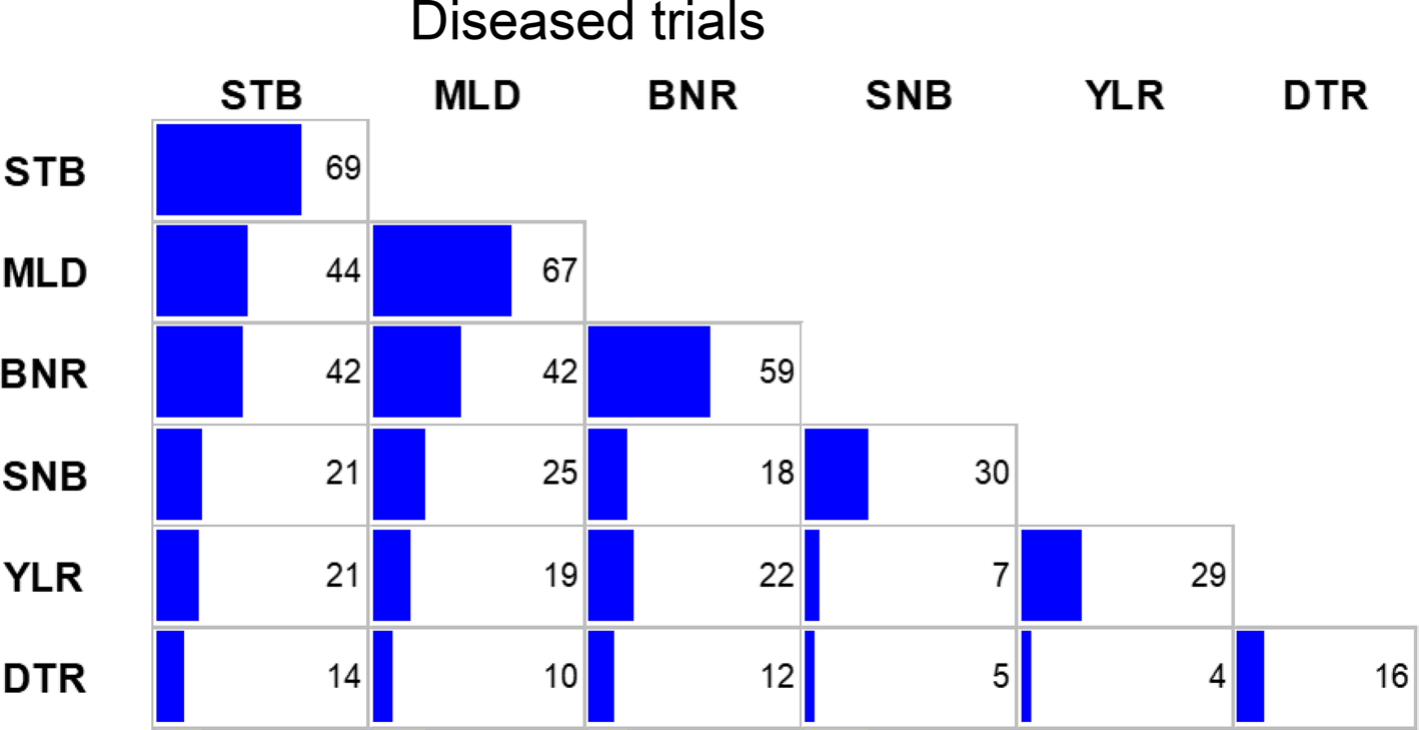
Diseased trials as percentage of total number of trials (*n *= 1755). The blue diagonal bars show the frequencies of individual diseases and the lower off-diagonal bars the joint occurrences of diseases in trials. The grey frame represents 100 per cent. *MLD* mildew; *BNR* brown rust; *STB* Septoria tritici blotch*; SNB* Septoria nodorum blotch; *YLR* yellow rust; *DTR* tan spot

In total, data from 1755 trials were available during the studied period 1983–2019; however, with respect to the diseases, not every trial was diseased, i.e. no disease indication was visible, as pointed out earlier in visual assessment of disease severity section. Figure 2 shows the frequencies of diseased trials relative to the total number of trials. STB was the disease occurring most frequently in trials with 69%, followed by MLD 67%, BNR 59%, SNB 30% and YLR 29%, whereas DTR was only available from 1996 onwards and occurred in 16% of trials. In 2% of the trials, none of the 6 diseases occurred, while in 1% of the trials all diseases showed scores greater than 1.

The susceptibility of a variety for a specific disease can only be assessed, if a symptom is visible. As shown in Fig. 2, the presence of the diseases varied from disease to disease and some diseases were only present in a small fraction of trials, e.g. YLR. Therefore, to determine the severity for a specific disease, we included only those trials in the analysis which showed an occurrence of this specific disease. However, for the prediction of yield by Model I and Model II all trials were included.

### Overall trend and change during 1983 and 2019

**Fig. 3:**
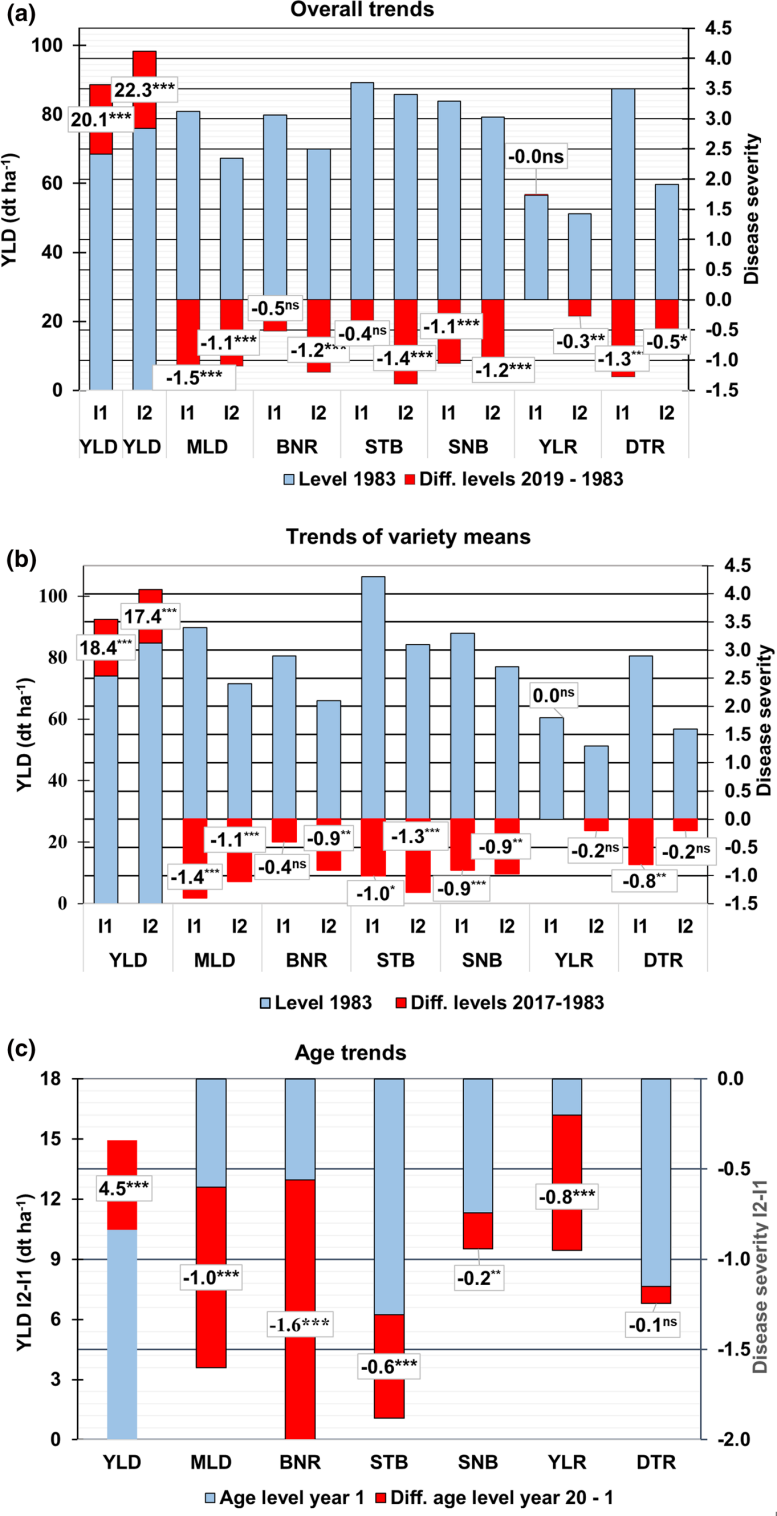
Trends and changes for **a** overall trends (using Eq. 2c), **b** variety means (using Eq. 3) and **c** age trends (using Eq. 6c). Blue columns refer to yield/disease levels 1983 (for DTR 1996) and levels for age year 1, red to the differences of levels 2019–1983 for overall trends, 2017–1983 (for DTR 1996) for variety trends and years 20–1 for age trends. Left y-axis represents the columns for YLD, right y-axis for diseases. Levels 2019 in **a** and 2017 in **b** are indicated by adding the blue and red columns considering the sign, prediction of age 20 in **c** is indicated analogously. *YLD* grain yield; *MLD* mildew; *BNR* brown rust; *STB* Septoria tritici blotch; *SNB* Septoria nodorum blotch; *YLR* yellow rust; *DTR* tan spot; *I1* intensity 1; *I2* intensity 2; ^ns^ non-signifcant; *significant at 5% level; **significant at 1% level; ***significant at 0.1% level

To evaluate the change of yield and disease responses during the study period, we estimated their levels in 1983 (for DTR in 1996) and 2019 using a quadratic regression function (Eq. 2a, b and c). The change is expressed as the difference between levels 1983 (1996) and 2019. We further tested whether the quadratic regression term was significant (Detailed results of the regression analysis are shown in Supplementary Material SM2, Table S1). In the overall trend, breeding progress and the influence of management and environmental factors are confounded. The results are shown in Fig. 3a. The blue columns represent the yield and disease severity levels at year 1983 (1996) and the red columns the change between 1983 (1996) and 2019. Figure 3b and 3c can be interpreted analogously. YLD I2 gained 22.3 dt ha^−1^ during 1983 and 2019, where the increase for I2 was 2.2 dt ha^−1^ higher. The yield difference between both intensities in 2019 was about 10 dt ha^−1^. The trend for YLD I1 showed a weak significant deviation from linearity, where for YLD I2 stronger nonlinearity was found.

Most trends for the diseases I1 and I2 followed a nonlinear pattern (Fig. 3a). The decrease of severity levels between 1983 (1996) and 2019 was notably larger and highly significant for I2, except for MLD and DTR, where for I1 only MLD, SNB and DTR showed significant reductions of −1.5, −1.1 and −1.3, respectively.

A significant decrease of severity of more than −1.0 was estimated for MLD I1 and I2, BNR I2, STB I2, SNB I1 and I2 and DTR I1. There was no change for YLR I1 and only a small one of −0.3 for YLR I2. A remarkable discrepancy in the overall trend for disease severity was found for STB. For this disease, a high decrease of −1.4 for I2 and a contrasting non-significant reduction of only −0.4 for I1 are present, i.e. no significant reduction in severity during 1983 and 2019 was achieved.

### Trend of variety susceptibility and change during 1983 and 2017

**Fig. 4:**
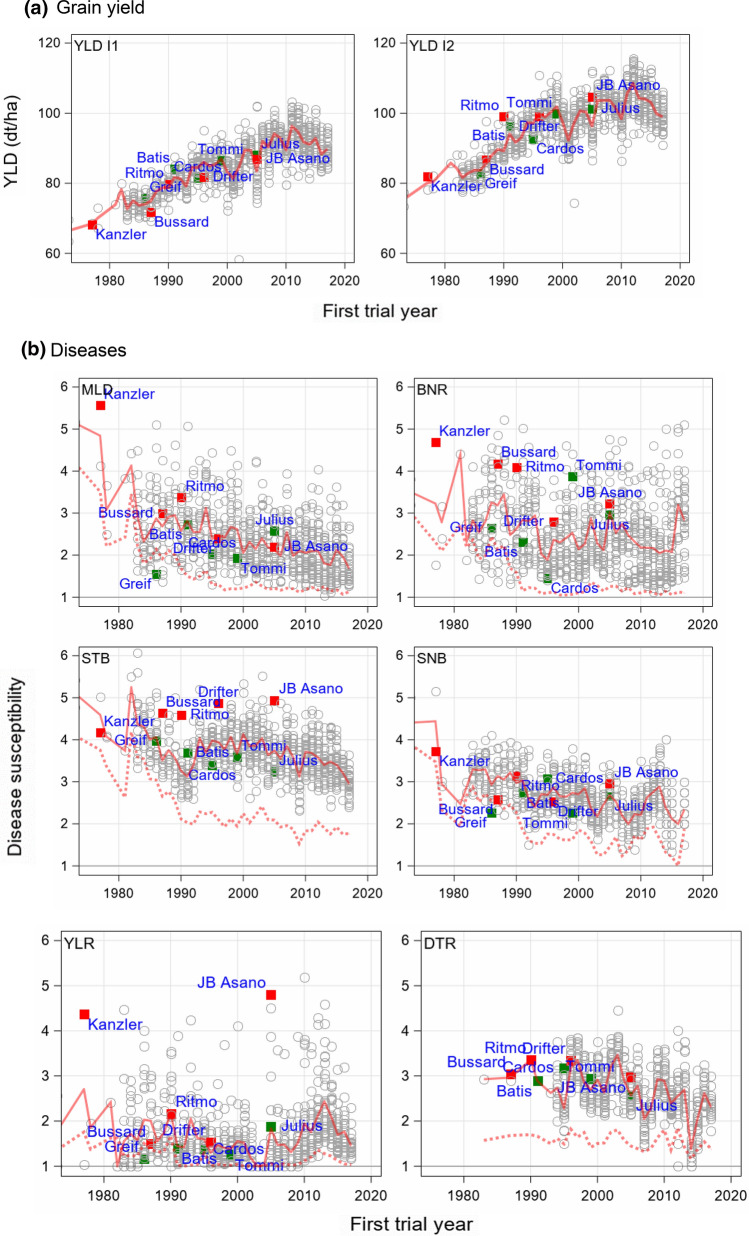
Yield means and disease susceptibility of varieties VSc (grey circles) plotted against first trial year of a variety for **a** YLD I1 and I2 and **b** diseases I1. VSc is given by the mean of the disease severity scores over all trials in which the respective variety was tested. Red solid lines represent the group mean of varieties with same first trial year for I1 and red dotted line for I2. The vertical difference between both lines depicts the treatment success obtained by fungicide and growth regulator application. Landmark varieties are represented by green markers for generally less susceptible and red markers for more susceptible varieties. *YLD* grain yield; *MLD* mildew; *BNR* brown rust; *STB* Septoria tritici blotch*; SNB* Septoria nodorum blotch; *YLR* yellow rust; *DTR* tan spot; I1 intensity 1

In Fig. 3b we show the breeding progress achieved for varieties by comparing yield and disease susceptibility levels for varieties between first trial year 1983 (1996 for DTR) and 2017. An orientation regarding a variety’s disease susceptibility is given by depicting landmark varieties in the plots of Fig. 4. Landmark varieties are popular varieties with considerable acreage during a longer period with well-known susceptibility characteristics. The varieties Kanzler, Bussard, Ritmo, Drifter and JB Asano were considered as generally more susceptible examples (red rectangles), where Greif, Batis, Cardos, Tommi and Julius as less susceptible ones (green rectangles). The varieties show mostly higher resistance or susceptibility levels to some diseases than other varieties, however, not an overall resistance or susceptibility. A more detailed description of the landmark varieties is given in Supplementary Material SM3, Table S2. Yield levels for varieties and the differences between levels given in Fig. 3b were derived by using Eq. (3) in the same way as described above for overall trends. However, it should be noted that the period for assessing the variety severity trend is a bit shorter (35 years) than for the overall trend (37 years). The effect of plant breeding progress and protection measures is demonstrated in Fig. 4 by comparing the trends for the treated (dotted red line) and the untreated intensity (solid red line).

Landmark varieties in Fig. 4a indicate the effect of plant protection against yield reduction. While for YLD I1 the resistant varieties (green markers) out-yielded the susceptible ones (red marker), for YLD I2, the susceptible varieties achieved higher yield than the more resistant ones, e.g. Drifter showed YLD below average for I1, where for I2, yield of Drifter was considerably above average. Nevertheless, the use of fungicides and growth regulators avoided yield reduction for both, the more susceptible as well as the more resistant varieties.

Generally, the trend pattern of the variety susceptibility was similar to the overall trend concerning linearity of trends. The yield levels of variety means with first trial year 1983 (1996) and 2017 did not deviate much from those of the overall trends (Fig. 3a and b) taking into account the shorter period between both levels for variety means.

Figure 4b shows a breeding progress towards varieties with lower disease susceptibility. The variety means represent a variety’s susceptibility observed under natural field infection conditions. Looking at the temporal development of disease susceptibility in more detail, a decreasing trend for I1 was estimated for all diseases except for YLR, indicating breeding progress towards more resistant varieties. Furthermore, for MLD, BNR, STB and SNB the difference between I1 and I2 was widening from about 1985 onwards where for DTR the difference became smaller. However, the 6 diseases showed diverse patterns. For STB I1 and I2, the susceptibility levels are highest among all diseases, with a large reduction for I2 and a smaller one for I1 (Fig. 4b). Moreover, fungicide treatment apparently did not completely control STB in I2. In I2, the susceptibility level remained at about 2, while in I1 the level was still between 3 and 4 in 2017. For STB, few varieties showed scores below 3 (Fig. 4b). Drifter and JB Asano are especially susceptible to STB. The plots for MLD and BNR revealed that disease severity was nearly completely controlled from 1990 onwards for BNR and from 1995 onwards for MLD. The susceptibility of varieties to YLR was generally low and nearly completely controlled by fungicides until 2010, and then the trend increased considerably, which can be attributed to the appearance of new virulent fungal races from Asia, especially “Warrior” and “Kranich”. Kanzler and JB Asano showed an extremely high susceptibility above 4 to YLR (Fig. 4b).

### Effect of variety ageing

**Fig. 5:**
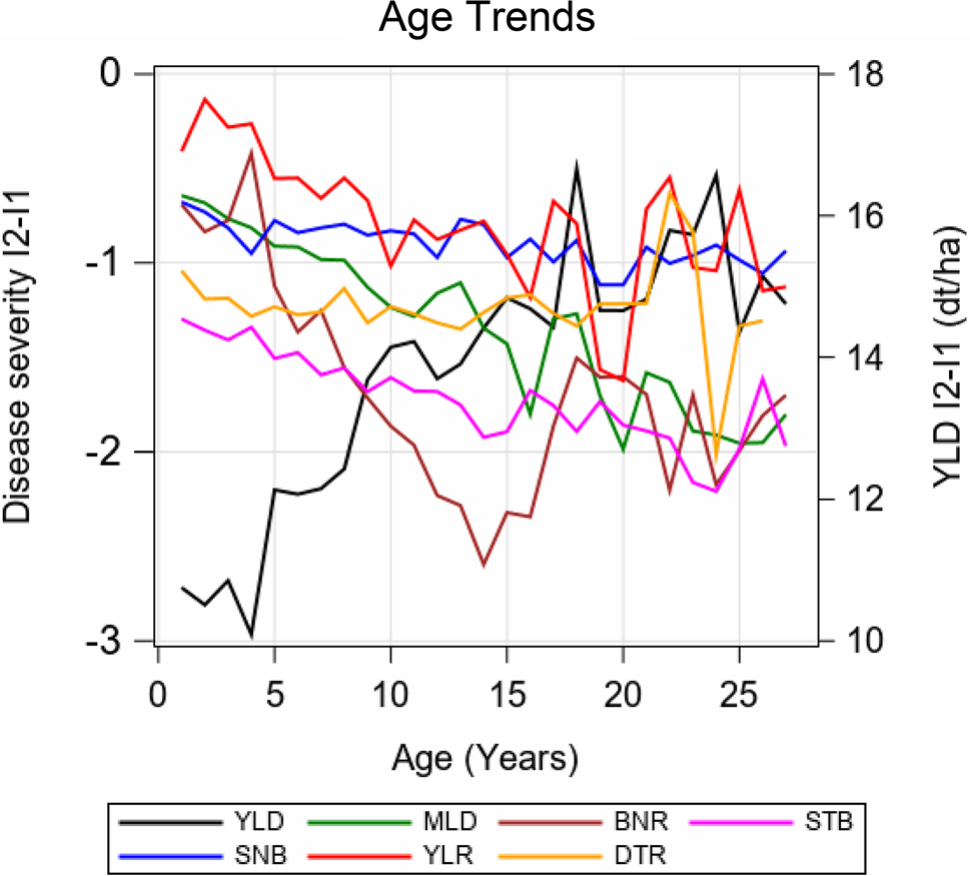
Adjusted group means of varieties belonging to the same age group based on absolute yield and disease differences I2–I1. Only varieties with four and more years of age are included. Adjusted means were derived by using $({\text{GY}})_{ikd} = D_{q} + ({\text{DH}})^{\prime}_{ikd}$ (Eq. 6c), where *D*_*q*_ is a fixed effect of varieties in the *q*th age group and $({\text{DH}})^{\prime}_{ikd}$ is a random deviation from the group mean. *YLD* grain yield; *MLD* mildew; *BNR* brown rust; *STB* Septoria tritici blotch*; SNB* Septoria nodorum blotch; *YLR* yellow rust; *DTR* tan spot; *I1* intensity 1; *I2* intensity 2

It is well known that the resistance of varieties towards some fungal pathogens may get lost partially or fully with increasing age of varieties due to population dynamics of fungal pathogens (Mackay et al. 2011). We modelled this effect by assuming that for I2 a variety’s loss of disease resistance is fully compensated by fungicide treatment, i.e. no ageing effect is present in I2. Therefore, we assumed that the difference of YLD I2–I1 and diseases I2–I1 over time are dependent on the stability of a variety’s resistance. If the resistance is getting weaker, we expect an increase in the yield and disease susceptibility differences. We evaluated only the 56 varieties with more than 3 trial years. The decay of yield and the increase of susceptibility were estimated as the contrast between the levels of the first and 20th trial year by a quadratic regression function (Eq. 5a and b). The results are shown in Fig. 3c and Fig. 5. The age trend for YLD I2-I1 was highly significant, reaching about 5 dt ha^−1^ during 20 years (red column) with an initial difference of about 10 dt ha^−1^ at age 1 (blue column). For BNR we found the strongest increase of disease susceptibility of −1.6, followed by MLD with −1.0, YLR with −0.8 and STB with −0.6. Disease resistance of STB is rather stable, however, showing a large divergence between I2 and I1 of about −1.3 at age 1. Only a small decrease of susceptibility for SNB and a non-significant decrease for DTR of −0.2 and −0.1 were estimated. It should be re-iterated that age trends represent the average of 56 varieties. Therefore, it may well be that some varieties showed a stable resistance to one disease and an unstable resistance to another one. Age trend of YLD reflects the overall stability of a variety, because in yield the effect on stability of all six diseases is accumulated.

### Impact of disease severity on yield (Model I)

By applying Model I we predicted yield I1 relative to I2 based on data of 2005–2019 comprising 645 trials. YLD I1 (%) indicates to which percentage the non-treated intensity I1 realized the yield level of the treated intensity I2 (100%). The relative difference of the predicted YLD I1 (%) to 100% can be considered as the yield reduction caused by diseases and lack of fungicides and growth regulators. In Table 3 the regression estimates of Model I are shown. Included in the selection procedure (see Supplementary Material SM1) were the covariates of the 6 diseases, where for BNR and STB a quadratic term was obtained. DTR was not selected. The average marginal variance (AMV) of Model I (Eq. 7a) was reduced by 13.6% compared to the basic model (Eq. 1a).

**Table 3:** Regression models for prediction of yield for years 2005–2019

Model I					Model II				
YLD I1(%) by field disease severity scores (Eq. 7a)					YLD I1 (%) by trial disease severity and variety susceptibility scores (Eq. 7b)				
Covariate	Estimate	Stderr	t-value	*p*-value	Covariate	Estimate	Stderr	*t*-value	*p*-value
*µ*	93.6030	0.7460	125.47	< .001	*µ*	99.7757	0.9788	101.94	< .001
*MLD*	−0.1338	0.0557	−9.23	< .001	*mld*^V^	0.7630	0.1888	4.04	< .001
*BNR*	0.2621	0.1041	1.28	0.199	*bnr*^V^	−0.3677	0.1300	−2.83	0.005
*STB*	0.5468	0.1410	1.86	0.063	*stb*^V^	−1.6524	0.2471	−6.69	< .001
*SNB*	−1.1590	0.1559	−3.51	< .001	*snb*^V^	0.3875	0.2564	1.51	0.131
*YLR*	−0.1183	0.0589	−19.67	< .001	*ylr*^V^	0.5078	0.1574	3.23	0.001
*BNR*^2^	−0.1333	0.0125	−9.49	< .001	*mld*^V^ ×* mld*^T^	−0.6214	0.0677	−9.18	< .001
*STB*^2^	−0.5144	0.0160	−8.34	< .001	*bnr*^V^ × *bnr*^T^	−0.4022	0.0331	−12.15	< .001
					*stb*^V^ × *stb*^T^	0.3003	0.1016	2.95	< .001
					*snb*^V^ × *snb*^T^	−0.4959	0.1312	−3.78	< .001
					*ylr*^V^ × *ylr*^T^	−0.9086	0.0712	−12.76	< .001
					*stb*^V^ × (*stb*^T^)^2^	−0.0659	0.0152	−4.34	< .001

*YLD* grain yield; *STB* Septoria tritici blotch; *MLD* mildew; *BNR* brown rust; *SNB* Septoria nodorum blotch; *YLR* yellow rust; I1 intensity 1; *mld*^T^, *bnr*^T^, *stp*^T^, *snb*^T^ and *ylb*^T^ denote disease

trial severity scores denoted by superscript *T*; *mld*^V^, *bnr*^V^, *stp*^V^, *snb*^V^ and* ylb*^V^ denote variety susceptibility scores indicated by superscript *V; I1* intensity 1

**Fig. 6:**
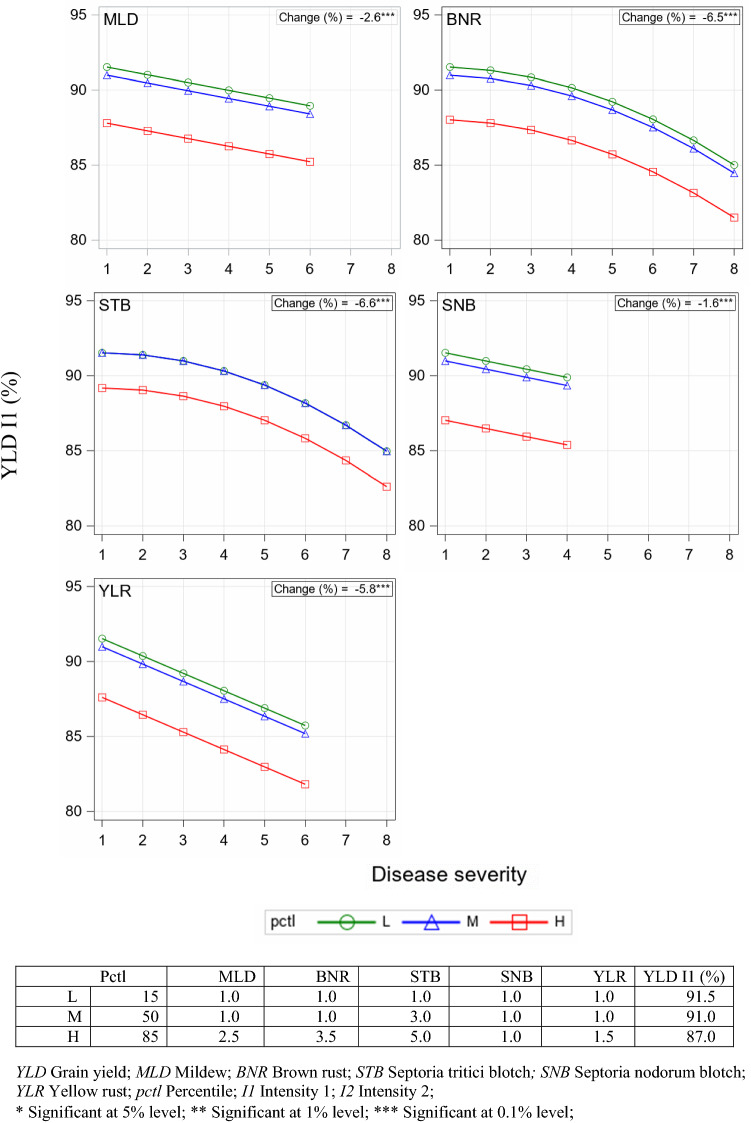
Predicted YLD I1 (%) by Model I (Eq. 7a) at 3 levels of disease severity plotted against disease severity scores between 1st and approximately the 99th percentile (pctl). L, M and H correspond to a low, medium and high disease severity for observations of all traits except the plotted trait

Figure 6 shows the decrease of YLD I1 (%) in response to the level of disease severity. The upper range of x-values varies between different diseases. The upper disease-specific severity level represents approximately the 99th percentile of the univariate distribution of disease severity for the specific disease on the 1–9 scale (see Supplementary Material SM4, Fig. S1). Three disease severity levels were considered, where “L” represents the low level at the 15th, “M” the medium level at the 50th and “H” the high level at the 85th percentile of the univariate distributions of disease severity (see legend of Fig. 6). The 15th percentile represents observations with no disease infection for all five diseases; this means that the predicted YLD at I1 of 91.5% corresponds to the yield level with no visible disease severity. The 50th percentile corresponds to predicted yield of 91.0%. For this percentile, only STB had a severity score of 3, whereas for the other diseases at least 50% of the observations had a score of 1. For disease severity above the 85th percentile the predicted YLD at I1 was less than 87.0%, i.e. for 15 percent of the observations a yield of less than 87.0% was predicted by Model I. For each disease we plotted the prediction function for the L-, M- and H-level. The 3 curves are parallel because Model I includes no interaction term between the linear covariates (Table 3). This means that the decrease in relative yield was identical for all three levels. The curves for the 15th and 50th percentiles were very close; for STB, they were identical. As shown in the table included in the caption of Fig. 6, the disease severity at the 15th and 50th percentiles was identical for all diseases, except for STB. This can be explained by the fact that in the dataset of Model I, the frequency of observations without disease severity was higher than 50%. We found frequencies with severity score of 1 (no disease) in the range of 53.8% (BNR) to 90.9% (YLR), except for STB with 23.2% (see Supplementary Material SM4, Fig. S1). The strongest yield decrease was predicted for STB with −6.6% and BNR with −6.5% in the severity range 1–8, followed by YLR with −5.8% (1–6). The decrease for MLD and SNB was much lower with values of −2.6% (1–6) and −1.6% (1–4), respectively.

### Impact of trial disease severity and variety disease susceptibility on yield (Model II)

**Fig. 7:**
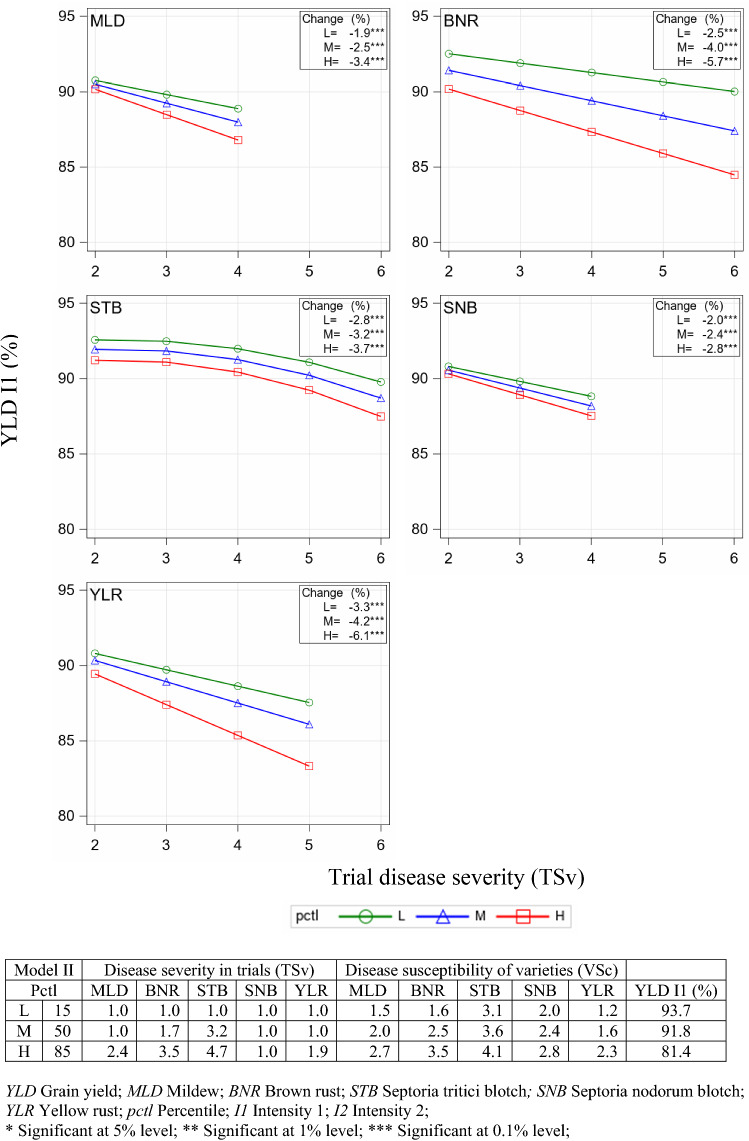
Yield I1 (%) for individual diseases predicted by Model II as a function of trials disease severity (TSv) and variety disease susceptibility (VSc) assuming a medium (M) level for overall TSv and VSc of non-plotted diseases, corresponding to 50th percentile (pctl). The maximum of TSv corresponds approximately to the 99th percentile. For each disease three lines, denoted by L, M and H, are plotted corresponding to 15th, 50th and 85th percentiles of VSc, marked by green, blue and red colour, respectively

By applying Model II, we assessed the impact of disease severity in a given trial TSv on yield under consideration of the disease susceptibility of varieties VSc. TSv was assessed as the mean of disease severity over all varieties in this trial (see Supplementary Material SM5, Fig. S2) and VSc as this variety’s mean of disease severity over all trials (see Fig. 4). In Table 3 the regression estimates of Model II are shown as the result of the selection procedure. Included are the five main effects for VSc, denoted by the superscript *V*, 5 linear-by-linear interaction effects of VSc and TSv denoted by the superscript *T*, and a quadratic interaction term for STB. The average marginal variance (AMV) of Model II (Eq. 7b) was reduced by 12.6% compared to the basic model (Eq. 1a). The intention of fitting Model II is to show the impact of increasing TSv on YLD at I1 (%) given 3 different levels of VSc. Figure 7 displays YLD at I1 (%) in response to TSv (x-axis), which ranges from score 2 to the score representing approximately the 99th percentile for the univariate distribution of TSv of each specific disease (see Supplementary Material SM6, Fig. S3). For all 5 diseases we found a significant interaction of TSv with the VSc levels, demonstrated by diverging lines in Fig. 7, especially for BNR and YLR.

The range of TSv scores was smaller as compared to the corresponding ranges for VSc scores in Model I, as TSv was derived from the trial-specific average severity scores over all varieties, which naturally reduces the maximum ranges. For varieties with low susceptibility level (L, green line), decreases were between -1.9% (MLD) and −3.3% (YLR), where for varieties with high susceptibility levels (H, red line) the decreases were higher and between −2.8% (STB) and −6.1% (YLR). Considering that the green line (L) represents the 15th percentile and the red line (H) the 85th percentile of the VSc distribution, this means that 15% of observations would show a yield reduction less than indicated by the green line, and in the other case, 15% would show higher yield reduction than indicated by the red line. The advantage of low vs. highly susceptible varieties becomes especially apparent when looking at plots for BNR and YLR. If TSv of BNR is increasing to 6, yield reduction of a highly susceptible variety is −3.2% larger as compared to a low susceptible one, i.e. that yield reduction of a low BNR-susceptible variety (−2.5%) is less than half of that of a highly susceptible one (−5.7%). Also, the yield reduction of a lowly YLR-susceptible variety (−3.3%) is only about half as compared to a highly susceptible (−6.1%) one. We found only a small interaction between VSc and TSv for SNB, MLD and STB.

## Discussion

The analysis presented here is based on observations from variety trials covering a large number of genotypes grown over a wide range of pedo-climatic conditions in Germany. The most important winter wheat diseases, originating from natural field infection, were evaluated. The fungicide and growth regulator treated intensity (I2) were compared with the untreated intensity (I1). Once again, we emphasize that in this study (1) diseases susceptibility of a variety describes the susceptibility observed on a set of field trials under natural infection, which may be lower than the potential severity assessed from artificial inoculation trials, and (2) diseases in I2 are not always controlled completely. We will first discuss the methodological aspects of the analysis of visual observations and then consider the results.

### Statistical aspects

#### Visual observations

All diseases were assessed by visual observations and scored on an ordinal 1–9 scale. Scoring was carried out by crop experts. Roughly speaking (and leaving aside the transition from the underlying quantitative scale to the assessed ordinal scale), this scale can be considered as the logarithmic transformation of the underlying percentage area of diseased leaves or spikelets. A score of 1 means that no disease symptoms were visible, corresponding to a severity of 0%, and a score of 9 that a severity of 62% to 100% was visible (Table 2). The precision of visual assessment may be subject to larger sampling errors than for metric measurements, such as yield. Further, if plots show a severity caused by 2 or more diseases, then it is difficult for the crop expert to discriminate the shares of individual diseases and assign appropriate scores. In consequence, it can be assumed that the precision of assessment is lower than for measurements; however, this is an appropriate and widely used method for diseases. On the other hand, the available data set entailed a vast number of observations with 34 and 37 years and an average of 47 trials per year, which ensured the reliability of results.

In fact, not every field trial showed an incidence for a specific disease, i.e. there were always trials in which all varieties had a score of 1. For the assessment of trends of a specific disease, we included only those trials for which a disease severity score for this particular disease was 2 or higher. We did so, because the disease susceptibility of a variety can only be investigated in trials with a visible disease symptom. As such, the six investigated diseases differed regarding their frequency of disease occurrence as shown in Fig. 2. For STB about 69% of trials were considered to be diseased, where for YLR only 29%.

#### Transformation vs. no transformation of visual observations on the 1–9 scale

We initially tried to evaluate breeding progress for diseases of I1 by dissecting genetic and non-genetic trends using the basic model (Eq. 1a) according to Piepho et al. (2014). The least square means for genotypes estimated under the assumption that the effect *G*_*i*_ for genotype *i* is fixed, plotted against a variety’s first trial year *r*_*i*_ gave negative means for BNR and YLR, and mean values less than 1 for MLD (see Supplementary Material SM7, Fig. S4). However, mean values below 1 are not interpretable. The reason for the occurrence of values below score 1 may be that the negative trend for the variety means (genetic trend) is biased downwards due to existing ageing effects (Piepho et al. 2014). In the previous section Effect of variety ageing, considerable ageing effects were found, especially for BNR, MLD and YLR. In this study we therefore did not estimate variety susceptibility by least square means, because variety susceptibility may be highly overestimated in the case of ageing effects. In a study on the effect of cultivar resistance estimated by least square means in French variety trials, Zhang et al. (2007) reported that for STB the resistance levels of varieties were overestimated, as compared to the experts’ classification without mentioning the possible reason. As they also used least square estimates from long-term disease observations, age effects may be responsible for their overestimated resistance of varieties.

To get least-square variety means in the range of the 1–9 scale, we transformed the observations by an empirical logistic function and then applied Eq. (1a) to estimate least square means in the transformed scale. Again, in order to obtain means in the 1–9 range, we back-transformed the means by the inverse logistic function. By this procedure negative means could be avoided, but we still obtained some means below score 1 (see Supplementary Material SM7, Fig. S4). Further, a remarkable discrepancy became apparent, when comparing the plots of the untransformed variety means (Fig. 4) with the corresponding back-transformed means (see Supplementary Material SM7, Fig. S4). Generally, for older varieties higher means were estimated indicating higher disease susceptibility on the back-transformed scale than on the untransformed scale, where for the more recent varieties very low susceptibilities were obtained in the back-transformed scale as compared to the untransformed ones. This means that the logistic transformation spreads the susceptibilities especially for MLD, BNR and YLR. This does not seem reasonable. We therefore decided not to apply a data transformation and not to estimate variety means by the least squares method based on Eq. (1a), because the results from untransformed visual disease observations provided a more understandable and realistic basis for interpretation. The same problems were encountered when expanding the basic model (Eq. 1a) to dissect long-term trends by including linear regression terms for modelling a genetic and a non-genetic trend component as described by Piepho et al. (2014). The main problem was a heavily biased genetic trendline for diseases under I1 falling below a severity score of 1 (YLR) and even below 0 (BNR) for recent varieties. We therefore did not use the model with dissection of trends. Instead, we estimated overall trends by Eqs. (2a, b, c and 3), where both components are confounded and derived the genetic trend based on simple untransformed variety means.

#### Analysing ordinal scores as metric data

In this paper, we analysed ordinal scores on a 1–9 scale as if they were metric data. This approach clearly constitutes an approximation as the assumptions underlying our linear mixed models cannot strictly be met. Diagnostic residual plots indicate, however, that no gross departures from assumptions of normality and homogeneity of variance were observed. Thus, we believe that our results are based on the best possible analyses, given the nature of the data.

There are, of course, several dedicated statistical methods for ordinal data that immediately come to mind as alternatives to our approach. Here, we briefly review our reasons for not using these approaches. (1) Shah and Madden (2004) review methods based on rank transforms. These methods require independently and identically distributed (i.i.d.) replicated observations per treatment and rely on approximate test statistics that require sample sizes not available for our data. Essentially, the means at our disposal correspond to unreplicated data and, as such, rank-based methods as reviewed in Shah and Madden (2004) are not applicable. (2) A second option is provided by so-called threshold models described, e.g. in McCullagh and Nelder (1989, § 9.2.4). This model assumes a multinomial distribution of the frequencies in the ordered categories. Again, these methods require i.i.d. replicate observations per plot (Thöni 1985), but our data are based on just a single observation per plot. (3) A further common suggestion is to collapse the ordered categories in just 2 compound categories and fit a binomial logit model. This approach suffers from the same problem as the ordinal threshold model because the sample size is equal to unity, and in this case the asymptotics for maximum likelihood estimation of the binomial model break down with complex linear models (Breslow and Lin 1995).

The conclusion at this point is that none of the seemingly obvious alternative routes of analysis are viable options for our data. All of them require a larger sample of i.i.d. observations per treatment (variety-by-environment combination), and we do not have such data. Instead, we have a single average score (usually based on 2 plots) for each treatment, and in this setting none of the proposed methods apply. In Supplemental Material SM8 we further explain why we are confident that the analysis we are performing, i.e. fitting a linear mixed model to the observed scores, is indeed the best option.

#### Selection procedure of Model I and Model II

Model I and Model II describe the association between the dependent variable YLD I1 (%) and independent variables (covariates) for the given data set. For both models we applied a forward selection procedure for the covariates (see Supplementary Material SM1).

Model I explained the association between disease severity scores and YLD I1 (%). Originally, we allowed for interaction terms between covariates after linear and quadratic terms were selected, e.g. the interaction between BNR and STB. Besides the linear and quadratic terms, nine interaction terms were finally selected. However, line plots for YLD at I1 (%) as function of disease severity scores with interaction terms revealed plots with non-interpretable pattern for some diseases caused by the interaction terms. The reason for the calculated abnormal pattern caused by interaction terms may be due to the frequency distribution of disease observations as shown in Supplementary Material SM4, Fig. S1. It may be that some cells in the frequency table have a high leverage on the selection of the interaction of 2 covariates. Other studies for predicting yield losses by linear models due to multiple diseases in winter wheat avoided inclusion of interaction effects (Zhang et al. 2007; Jahn et al. 2012 We therefore did not include interactions in the selection process, because “If interpretability of a statistical model is of relevance, simplicity must be kept in mind” (Heinze et al. 2018). We believe that interpretability of the regression model is more important.

Model II explained the association between YLD I1 (%) and VSc and a TSv. In this model, we consider the interaction between the covariates VSc and TSv. However, both covariates are derived from the same disease. This means that the interaction of disease covariates in Model I and in Model II is of a different nature. In Model I it is the interaction between covariates of 2 different diseases; therefore, for Model II, we did not get the same interpretation problems of plots as for Model I. In Model II, main effects for the covariates were included even if they did not increase *R*^2^, if the corresponding interaction effect was included later. It is indispensable to include main effects of covariates if interaction terms are included in the model, as this ensures a fit that is invariant to linear transformation of the covariates (Nelder 2000).

Not to include interactions terms of covariates between diseases in Model I and Model II is supported by results reported by Miedaner et al. (2014, 2020), who detected no substantial correlations among individual disease responses in wheat between MLD, STB and YLR, indicating that the resistances are independently inherited; in particular, they found that STB and YLR do not interact in wheat. Further evidence of a weak association was indicated by estimating the correlation coefficients of pairwise correlations between 789 variety means for the 6 diseases. After removing the time trend, the Pearson partial correlation coefficients indicated an average weak association of *r* = 0.26 in the range *r* = 0.49 (BNR x DTR) to 0.00 (STB x SNB), respectively (data not shown). Further evidence of a weak association between diseases provides Supplementary Material SM4, Fig. S1. Model II is, as Model I, additive with respect to the covariates, and in consequence this means that yield reductions caused by individual diseases adding up.

### Application of fungicides and growth regulators

In Fig. 1 we have seen that the treatment frequency index (TFI) reached nearly 3 until 1993, then dropped to less than 2 in 2003 and then increased again to about 3 in 2016. We found no clear causes for the reduction in TFIs from 1994 onwards until 2013. An increasing share of disease-resistant varieties, a reducing level of nitrogen application and presumably general policy efforts aiming for reduced fungicide use may partly explain the temporally low TFIs. The increase of TFI after 2013 can mainly be explained by the epidemic occurrence of new YLR races (“Warrior” and “Kranich”). Many varieties were susceptible to these new races, which necessitated higher fungicide treatment intensities.

No significant difference in the intensity of fungicide treatment is seen, when comparing the VCU trials used in the present study with actual on-farm practice. In the network of reference farms for plant protection, TFIs were recorded annually during 2007 and 2017 from wheat-growing farms, which are representative for the wheat-growing areas in Germany (Dachbrodt-Saaydeh et al. 2020). The average fungicide TFI during 2007 and 2017 was 2.2 (1.8–2.7) for on-farm conditions and 2.3 (2.0–2.9) for VCU trials at I2. The average growth regulator TFI was 1.0 (0.8–1.1) on-farm and 1.0 (0.9–1.1) for VCU. Hence, the chemical treatment intensity in the VCU trials is comparable to on-farm practice.

### Overall trends and trends of variety means

The overall trends represent the confounded genetic and non-genetic effects. For I2, additionally to the genetic effects, the impact of fungicide and growth regulator treatment are driving factors for the changes found in the overall trends. Figure 3 shows a gain from selection for all diseases resulting in reduced susceptibilities of varieties in both I1 and I2, except for YLR under I1. The increase of YLR susceptibility from 2013 on (Fig. 4) can be explained by the occurrence of the novel “Warrior” and “Kranich” races. For a long period YLR was a pathogen which was epidemic only every 5–10 years. Supplementary material SM5, Fig. S2 shows a strong severity for 1989, 1999–2001 and especially from 2013 onwards due to the new, more temperature-tolerant races (Milus et al. 2009); in recent years, YLR has been occurring epidemically every year.

The susceptibility trends of varieties shown in Fig. 4 were not decreasing uniformly. When comparing the trend lines of I1 and I2 for variety means plotted against a variety's first trial year, it becomes apparent that until about 1990 the susceptibility levels of varieties for I2 decreased considerably and then slowed down (e.g. STB) or levelled off (e.g. MLD, BNR). For I1, a similar line pattern is evident until 1990 (Fig. 4), but at a higher level. After 2000 a moderate decrease (e.g. MLD) or even no change (e.g. STB) occurred. The susceptibility levels generally decreased strongly until about 1990, presumably due to progress in resistance breeding. Widening crop rotations, an essential factor in IPM, by avoiding wheat-after-wheat can help to reduce DTR occurrence significantly (Mazzilli et al. 2016). The fungicide application rate (TFI) was considerably higher before 1990 than afterwards (Fig. 1). Hence, insufficient disease control in the early years is likely of minor relevance. Presumably, improved efficacy of fungicides exerted a major influence for a better disease control in I2 after 1990.

Comparing the breeding progress towards more resistant varieties from long-term field trials under natural infection (the present study) with results from trials with historic varieties or trials with artificial inoculation may be problematic. Ahlemeyer and Friedt (2011) assessed susceptibility of 90 winter wheat varieties registered between 1966 and 2007 from field trials grown at five sites over 3 years during 2012–2014 with 2 intensities. For the intensity without fungicides they found a linear decreasing trend of variety susceptibility for MLD, BNR and STB over registration years. To compare this finding with our study we scaled down the changes (42 years) to the period of our study (35 years). In their study a considerable higher decrease level compared to our results was reported (−2.7 versus −1.3 for MLD, −1.2 versus −0.5 for BNR and −1.0 versus −0.7 for STB). At this point the question arises, which study better represents actual breeding progress: the progress derived from vintage trials with historical varieties (Ahlemeyer and Friedt 2011) or the progress found in a study with long-term historical data as used in the present study. We suggest a new perspective on this question: the trend in breeding progress for diseases in winter wheat varieties under natural infection can be thought of as being composed of an invisible and a visible component. The invisible component can be considered as the progress achieved by keeping the resistance level of new varieties constant under a dynamic race spectrum or we could say “breeding for stabilizing resistance”. The visual component can be attributed to the absolute decrease of the susceptibility level of new varieties that occurs additionally to the invisible component, or we could say “breeding for improved resistance”. The extent of the invisible effect can be seen when comparing the change for MLD, BNR and STB as reported by Ahlemeyer and Friedt (2011) for historic varieties in vintage trials with the change of the corresponding diseases in the present study (Fig. 3b). The difference between both may be considered as the invisible progress. In other words, the total breeding progress is the progress measured by historical varieties grown under the recent pathogen and race situation and the visible breeding progress is the progress measured by long-term trends of historical data, as given in the present study.

In another study of Zetzsche et al. (2019) relative disease susceptibility for several races of YLR and BNR was assessed by artificial inoculation of 199 European winter wheat varieties registered between 1966 and 2013. For both diseases, the decrease of susceptibility was found to be linear and much higher than in our study. As the present study is based on natural infections, differentiation among varieties is lower than in artificially infected trials. This is caused by the irregular occurrence of some diseases (Fig. 2) and the generally lower disease severity, which hampers the differentiation between resistant and moderately susceptible varieties. Only highly susceptible varieties clearly show their “weakness” also under (low) natural infection. This is substantiated by the means of highly susceptible varieties like Kanzler for all diseases and Ritmo for BNR, STB, SNB and DTR (Fig. 4). We want to stress that the long-term trends of disease susceptibility found in vintage trials with historic and current varieties are difficult to interpret due to possible changes in the dynamics of the race spectrum. Breeding progress of disease susceptibility assessed for historic varieties may be strongly overestimated. We believe that the breeding progress expressed as the change in disease susceptibility from historic long-term data provides a more realistic picture, because it assesses the susceptibility of varieties under their current pathogen and race spectrum (i.e. the spectrum at the time of their release and/or widespread cultivation) under natural infection and other environmental conditions.

### Effect of variety ageing

It is well known that resistance genes of varieties get ineffective against some pathogens partially or fully due to changes in the pathogen populations, depending on the resistance genes accumulated in the genome and the virulences of the race spectrum (e.g. Zhang et al. 2007; Mackay et al. 2011). For the evaluation of the age effect by the difference of disease severities at I2 and I1, we assumed that no full control of fungal disease was achieved in I2, but rather that fungicide application compensates yield loss only partially. We therefore assumed that fungicide application hides ageing of varieties due to loss of resistance. Admittedly, under I2 some varieties may show an ageing effect. This means that we estimated the difference between ageing effects at I2 and I1, which may be smaller than the ageing effect of I1. However, the ageing effect of I2 and I1 may be small compared to the effect of I1.

In Figs. 3c and  4 we have shown that the basic level (age year 1) of differences for individual diseases varies over a wide range of −0.2 (YLR) to −1.3 (STB). Presumably, this depends on the one hand on the susceptibility level of varieties, which is highest for STB, but on the other hand it may be influenced by the efficacy of fungicide treatment against a specific disease; thus, e.g. if efficacy is higher, then the difference may become larger. Further, Fig. 4 shows that the volatility of age trends is increasing with age due to the decreasing number of varieties with higher age as can be seen for example for MLD and YLR. The reduction in YLD after 20 years, estimated in the present study, is low compared with a linear annual increase by 0.8 dt ha^−1^ reported by Mackay et al. (2011) for UK winter wheat variety trials 1948–2007. This corresponds to a total yield reduction of 16 dt ha^−1^ after 20 years, which is more than three times the reduction we estimated. The reason for this large discrepancy may be that in the UK study older varieties with higher susceptibilities were included.

Age trends clearly show that the durability of resistances is widely differing between diseases. For SNB and DTR the age trend is absent or only minimal. For SNB, this confers with the quantitative inheritance of resistance and the absence of race specificity in the pathogen population. For DTR, where both qualitative and quantitative resistances are reported (Singh et al. 2019), this means that in the German varieties, preferably the latter are included. Because quantitative resistances are caused by many small quantitative trait loci (QTL) scattered across the whole genome, their durability is high and the fungi are not able to adapt to those resistances within a short time frame (McDonald and Linde 2001). The opposite is true for MLD and BNR. MLD susceptibility increases successively with a higher age of the varieties. Also, for BNR, a sharp decline can be seen till age 15, while afterwards an increase in resistance was found. The reason is that only 5 varieties are older than 15 years with relatively stable resistances. MLD and BNR are mainly inherited by qualitative resistances that can be overcome by the pathogens by selection of virulent isolates. For example, Tommi suddenly became susceptible in 2006 when the *Lr*37 gene was overcome by virulent isolates (Serfling et al. 2013). Varieties that remain resistant for their whole lifetime, like Tommi for MLD, still have an effective resistance gene, or their resistance is based on quantitative resistances, which exist in both diseases. For YLR, some resistances changed after 2011 with the advent of the “Warrior” race, for example Elixer, Diskus and Matrix, where other cultivars remained highly resistant so far, like Tobak and Anapolis. It should be considered that also the relative importance of pathogen populations changes over time, also subject to an ageing effect. In the beginning of the trials SNB was considered a very important disease with a lot of resistance breeding efforts. Since 2006 the occurrence of SNB dropped considerably. In the last 4 years the disease occurred only in one or two locations every year, but STB is now considered as the most important leaf disease in wheat. Zhang et al. (2007) reported that in France SNB was no longer an important disease in wheat already since the 1990s. According to Shaw et al. (2008), who analysed this population change in the UK, the *Zymoseptoria tritici* causing STB is more tolerant to hot, dry weather episodes in summer and favoured by lower SO_2_ emissions in the atmosphere, while for *Parastagonospora nodorum* the opposite is true. Also, within biotrophic pathogen populations, the combination of virulences changed and also the aggressiveness, i.e. the number of spores a virulent isolate is able to produce. Our results confirm STB as the most important leaf disease in winter wheat at present. It occurs most frequently and has the highest severity level in I1 and a low breeding progress estimated in I1. On the other side, it shows the smallest age effect and the highest effect due to disease control measures (overall trend). Again, the “Warrior” race of YLR is more virulent than the “old” European races, i.e. it can knock out more resistance genes, and has a shorter latency period and a higher spore production rate, both contributing to a higher aggressiveness (Hovmøller et al. 2016). Therefore, the “old” races nowadays do not occur any more (GRRC 2019). This also affects the resistance portfolio of the released wheat varieties and it is to be expected that yellow rust will now appear regularly in all regions of Germany.

### Model I and II

For Model I and Model II we chose the relative yield to model yield reduction, because several authors reported that absolute yield reduction (dt ha^−1^) was positively correlated with treated yield (e.g. Zhang et al. 2007). The correlation is caused by a joint time trend in YLD I1 and YLD I2 as can be seen in Fig. 4a when comparing time trends. For both models, the time trend *t*_*k*_ was not selected as a covariate in the model selection process because it was removed by dividing YLD I1 by YLD I2. From Fig. 4b it has been shown that disease occurrence in I2 was not fully controlled by fungicide treatment. We therefore considered the yield difference I2–I1 as yield reduction rather than yield loss. Moreover, yield loss is generally defined as the difference between the attainable yield and the actual yield (Zadoks, Schein 1979, p. 246).

#### Model I

Model I explains the impact of multiple disease severity on YLD I1 relative to I2 by an additive model with linear regression coefficients for MLD, SNB and YLR, but with quadratic coefficients for BNR and STB (Table 3). Additivity means that YLD I1 (%) predicted by a specific disease is independent from the other diseases. Yield reduction was predicted for all diseases in response to disease-specific severity scores. To account for differences between diseases with regard to their maximum observed severity, we always displayed the disease severity between score 1 and the maximum observed score, i.e. approximately to the 99th percentile of each specific disease. The 99th percentile for BNR and STB reaches a score of 8, whereas that for SNB is considerably lower at a score of 4 (Supplementary Material SM4, Fig. S1). Diseases caused a yield reduction in descending order of STB, BNR, YLR, MLD and SNB (Fig. 6).

For Model I, YLD I1 (%) was predicted as 91.5% if no disease indication was visible for all 5 diseases, corresponding to a yield difference of 8.5% to the YLD I2 (see legend Fig. 6). The yield difference is likely deriving from the effect of growth regulators plus a yield enhancing effect of the application of some fungicides (i.e. Strobilurins) leading to a delayed leaf senescence and longer green leaf duration (Ballini et al. 2013; Schierenbeck et al. 2019; Wu and von Tiedemann 2001). It should be noted that according to the general principles of integrated crop protection, which are mandatory for all EU member states since 2014 (BMEL 2010), the application of fungicides is restricted to the protection of crops against fungal diseases. Fungicides must not be applied in the absence of disease symptoms. Additionally, other diseases, which were not considered in the analysis, like Fusarium head blight (*Gibberella zea*), may also have contributed to the yield difference.

In the literature, numerous results from studies on yield loss in winter wheat caused by fungal diseases are reported arising from rather heterogeneous experiments and data sources. Some studies are based on artificial infection (e.g. Zhang et al. 2006 and 2007), others on only a few varieties (e.g. Bhathal et al. 2003), years (e.g. Loyce et al. 2008) or locations (e.g. Jevtic et al. 2017). Thus, their results vary considerably and are not directly comparable among each other and with the present study. Generally, STB, BNR and MLD were reported to occur more frequently compared to other fungal diseases, e.g. YLR occurred less frequently but was epidemic, which is in line with the results of this study (Fig. 2). Jahn et al. (2012) reported on results from the German plant protection service 2003–2008. They found an average yield loss due to STB of 4.8 dt ha^−1^, to BNR of 2.5 dt ha^−1^ and to MLD of 1.5 dt ha^−1^ (YLR and SNB were not reported); however, the relative magnitude does not match with our results.

Savary et al. (2019) reported yield losses in winter wheat for Northwest Europe, including Germany, compiled from an expert-based survey in 2016/2017. Among fungal pathogens, the survey estimated yield losses of 5.8% (5.8%) for YLR, 5.5% (6.6%) for STB, 2.5% (6.5%) for BNR, 2.2% (2.6%) for MLD and 0.1% (1.6%) for SNB (in brackets results from our Model I). Results for YLR, STB and MLD are about comparable, but for BNR and SNB we predicted a higher reduction than reported from the survey. These differences may partially be due to small sample sizes available in the survey of Savary et al. (2019), which ranged from 2 to 15 responses per disease.

Yield reductions due to individual diseases shown in Fig. 6 seem to be rather low compared with potential losses reported from other studies. Singh et al. (2016) referred to potential yield losses for YLR of 5%–50%, MLD of usually less than 10% and STB of 30%–50%. The results obtained in this study, however, need to be considered not as potential in the sense of maximum losses, but as average reductions for a specific disease at given severity level. In the case of BNR this means, given a severity score of 8, that we can expect an average yield decrease of 6.5%. In some trials with severity score 8, however, the reduction may be considerably higher or lower. We further should point out that yield reductions are predicted under multiple disease severity, which means that yield reductions are additive.

In this context, it further needs to be noted that disease severity was not controlled completely by fungicide treatment in I2 as indicated by the disease susceptibility of varieties shown in Fig. 4. Average susceptibility levels for STB and SNB were markedly above 1. This could be caused by the fact that due to the experimental setup all varieties were treated at the same time, which was most likely not the optimal time for every variety. We therefore investigated whether the residual disease severity observed at I2 had a verifiable impact on YLD at I2 and conducted the same model selection procedure as for the relative yield at I1 by Model I. The result of the selection procedure showed that only the covariate time *t*_*k*_ (years) reduced the *R*^2^ by 5.4%, whereas no disease had a reducing effect. This indicates that fungicide treatment controlled disease severity in I2 to such a degree that the inclusion of the diseases as covariates brought no further improvement of *R*^2^.

#### Model II

By Model II the impact of a variety’s disease susceptibility, or conversely, its resistance level, on yield reduction was evaluated under a wide range of trial disease severity levels. We found that the yield reduction of a highly versus low susceptible variety may be more than twice as high (Fig. 7), indicating the benefits of breeding for more resistant varieties. As minimum value for the x-axis (TSv), we used 2, because TSv score 1 means absence of the disease; hence, an interaction is only realistic if a TSv is present.

All diseases showed interaction, however, with different effects on the magnitude of yield reduction. Large effects were predicted for BNR and YLR and small ones for SNB. Increasing TSv caused considerable yield reduction for BNR and STB; however, between both diseases a remarkable difference in yield reduction became apparent with respect to VSc level L and H. The difference for BNR accounts for 3.2%, where for STB it reaches only 0.9% between L and H. This can mainly be attributed to the wider spread of VSc for BNR as compared to STB. The susceptibility level between the 15th and 85th percentile for BNR is in the range of 1.6–3.5, but for STB it is considerably smaller (3.1–4.1, see legend Fig. 7). Consequently, a variety’s resistance potential to reduce yield for BNR is larger than for STB, despite the fact that yield reduction for STB under high TSv is large.

## Conclusions

Significant progress has been made in raising grain yield and in decreasing disease susceptibility of winter wheat varieties during 1983–2019. Progress for the treated intensity (I2) was considerably higher than for the untreated intensity (I1) due to the interaction effect of fungicide, growth regulators and variety ageing with genotypes. With increasing age of varieties, a significant yield decrease and increase of disease susceptibility occured, indicating that successful breeding entails two key steps; first, to retain resistance of new varieties under current race composition (invisible part), and second, to further improve resistance (visible part). Mixed linear regression models are an effective approach to determine yield reduction in long-term trials with multiple disease severity using diseases as covariates. Model evaluation as well as correlation of diseases indicated that the impact of individual diseases can be considered as being additive, i.e. independent. The largest yield reduction was predicted for STB, BNR and YLR, moderate ones for MLD, but minor ones for SNB. For all diseases, interaction between disease susceptibility of varieties (VSc) and trial disease severity (TSv) became apparent, especially for BNR and YLR. The functional relations between disease severity and yield reduction found in this study may provide helpful empirical evidence to support the present discussion on EU-policies towards reducing pesticide use and its environmental impacts. With reduced availability of chemical plant protection, the importance of resistance breeding and the role of the testing authorities are going to further increase in the future. Improved integration of expertise of (or improved cooperation of) breeders and plant pathologists will be required to better handle the upcoming challenges. Our study clearly shows that resistance breeding is a continuous challenge caused by rapidly evolving plant pathogen populations as illustrated by the described ageing effects. The speed of these changes might even increase for thermophilic pathogens with the climate change we are facing. In future, a continuous monitoring of the most relevant pathogen populations in terms of their virulence and molecular signatures (“pathogenomics”) would be important for early detection of population changes that are relevant for the breeders. In parallel, resistant cultivars should be analysed genetically on a regular basis to provide an array of different resistance sources and thus improve durability.

## Electronic supplementary material

Below is the link to the electronic supplementary material.

Supplementary file1 (PDF 108 kb)

Supplementary file2 (PDF 289 kb)

Supplementary file3 (PDF 28 kb)

Supplementary file4 (PDF 46 kb)

Supplementary file5 (PDF 203 kb)

Supplementary file6 (PDF 92 kb)

Supplementary file7 (PDF 268 kb)

Supplementary file8 (PDF 1219 kb)
