# Supplementary material for: Breeding progress of disease resistance and impact of disease severity under natural infections in winter wheat variety trials
Source: Theor Appl Genet. 2021 Mar 13;134(5):1281–302. doi: 10.1007/s00122-020-03728-4 (PMC8081715; doi:10.1007/s00122-020-03728-4)
Supplement: Supplementary file 1 — Supplementary file1 (PDF 108 kb) [file 122_2020_3728_MOESM1_ESM.pdf]

Supplementary Material SM1

Selection procedure for covariates in Model I (Eq. 7a) to predict impact of natural disease severity on yield

Covariates to be tested for inclusion are t_k, *MLD, BNR, STB, SNB* and *YLR, DTR*.

First stage: Add the first covariates t_k to Eq. (2b), the second *MLD*, third *BNR*, …last *YLR*, include covariates with highest coefficient of determination R^2 for mixed models (Piepho, 2019) in the model, calculate R^2 of each of the remaining covariates, include covariate with highest R^2 as second covariate in the model continue result: *MLD, BNR, STB* , *SNB, YLR, DTR*

Second stage: add first quadratic term to the selected linear terms in the first step
add second quadratic term to the selected linear terms in the first step
follow same procedure for quadratic terms as in first and second step
result: STB^2 and BNR^2

Final model: $E(z_{ijk}) = \mu + \alpha_1 MLD_{ijk} + \alpha_2 BNR_{ijk} + \alpha_3 STB_{ijk} + \alpha_4 SNB_{ijk} + \alpha_5 YLR_{ijk} +$

$$+\beta_1 BNR_{ijk}^2 + \beta_2 STB_{ijk}^2.$$

The coefficient of determination for the final model is R^2=13.4.
