# Supplementary material for: Breeding progress of disease resistance and impact of disease severity under natural infections in winter wheat variety trials
Source: Theor Appl Genet. 2021 Mar 13;134(5):1281–302. doi: 10.1007/s00122-020-03728-4 (PMC8081715; doi:10.1007/s00122-020-03728-4)
Supplement: Supplementary file 2 — Supplementary file2 (PDF 289 kb) [file 122_2020_3728_MOESM2_ESM.pdf]

Supplementary Material SM2

In this Supplementary Material, we show the estimated regression coefficients of the mixed model quadratic regression analysis described in the Statistical Analysis sections 2.4.2 – 2.4.7.

Overall trends (Table S1a):

The fixed term of the model for overall trend I1 and I2 is given by

$$\mathrm{E}(y_{ijkl}) = P_l + \alpha t_k + \beta t_k^2$$ for yield (Eqs. (2a), (2c)), and by

$$\mathrm{E}(y_{ijk}) = \mu + \alpha t_k + \beta t_k^2$$ for diseases (Eqs. (2a), (2b)).

Note: For numerical reasons, the regression coefficients shown in Table S2a are calculated by $t_k - 2000$, the centered time covariable t_k;

Yield and disease levels 1983 (1996 for DTR) and 2019 are the regression estimates of the expected values $\mathrm{E}(y_{ijkl}|t_k = 1983)$ and $\mathrm{E}(y_{ijkl}|t_k = 2019)$ for yield and $\mathrm{E}(y_{ijk}|t_k = 1983)$ and $\mathrm{E}(y_{ijk}|t_k = 2019)$ for disease. The change is the difference between levels 2019 and 1983 (1996 for DTR).

Trends for variety means (Table S1b):

Trends of variety means are calculated from the means of individual varieties over all trials in which the variety was present. The fixed term of the trend model for I1 and I2 is given by

$$E(y_i) = \mu + \alpha r_i + \beta r_i^2,$$ (Eq. (3)).

Note: For numerical reasons, the regression coefficients shown in Table S2b are calculated by the centred covariable $r_k - 2000$, where r_k is the varieties first trial year.

Yield and disease levels 1983 and 2017 are the regression estimates of the expected values $\mathrm{E}(y_i|r_i = 1983)$ and $\mathrm{E}(y_i|r_i = 2017)$. The change is the difference between levels 2017 and 1983.

Trends for variety age (Table S1c):

Trends for variety age are calculated from the expected values of the difference between observations I2 – I1 given by

$$E(d_{ijkl}) = \Delta P_l + (-\delta_1^1)a_{ik} + (-\delta_2^1)a_{ik}^2$$ for yield (Eq. (6a)) and

$$E(d_{ijk}) = \Delta\mu + (-\delta_1^1)a_{ik} + (-\delta_2^1)a_{ik}^2$$ for diseases (Eq. (6b)), where the regression covariable $a_{ik} = (t_k - r_i)$ represents the age of variety i in year k.

Yield and disease levels for age year 1 and 20 are the regression estimates of the expected values $\mathrm{E}(d_{ijkl}|a_{ik} = 1)$ and $\mathrm{E}(d_{ijkl}|a_{ik} = 20)$ for yield and $\mathrm{E}(d_{ijk}|a_{ik} = 1)$ and $\mathrm{E}(d_{ijk}|a_{ik} = 20)$ for disease. The change is the difference between levels 20 and 1.

Table S1a Estimates of regression coefficients for overall trends

		Overall trends 1983 - 2019													
		Regression coefficients I1				Regression coefficients I2				Levels I1			Levels I2		
		P_1	P_2/μ	α	β	P_1	P_2/μ	α	β	1983	2019	Diff	1983	2019	Diff
YLD	Est	83.0283	84.9479	0.5410	-0.0183	95.7066	94.8437	0.6981	-0.0271	68.5418	88.6196	20.0778	76.0038	98.3192	22.3154
	SE	1.5328	2.7664	0.1446	0.0085	1.6884	2.9749	0.1540	0.0090	2.3857	2.5660	3.0527	2.5709	2.7631	3.2525
	PT	0.0000	0.0000	0.0002	0.0313	0.0000	0.0000	0.0000	0.0027	0.0000	0.0000	0.0000	0.0000	0.0000	0.0000
MLD	Est		2.2237	-0.0427	0.0006		1.3058	-0.0337	0.0016	3.1218	1.6285	-1.4933	2.3422	1.2431	-1.0991
	SE		0.0943	0.0050	0.0005		0.0539	0.0030	0.0003	0.1489	0.1733	0.1809	0.0852	0.1038	0.1077
	PT		0.0000	0.0000	0.2225		0.0000	0.0000	0.0000	0.0000	0.0000	0.0000	0.0000	0.0000	0.0000
BNR	Est		2.1714	-0.0184	0.0020		1.1732	-0.0380	0.0023	3.0584	2.5387	-0.5196	2.4936	1.2942	-1.1994
	SE		0.1371	0.0084	0.0008		0.0725	0.0044	0.0004	0.2569	0.2427	0.2907	0.1333	0.1273	0.1516
	PT		0.0000	0.0277	0.0164		0.0000	0.0000	0.0000	0.0000	0.0000	0.0739	0.0000	0.0000	0.0000
STB	Est		3.8255	-0.0098	-0.0012		2.2029	-0.0433	0.0016	3.6399	3.1986	-0.4414	3.3905	1.9428	-1.4477
	SE		0.1008	0.0071	0.0006		0.0884	0.0062	0.0006	0.2327	0.1763	0.2367	0.2006	0.1594	0.2087
	PT		0.0000	0.1654	0.0582		0.0000	0.0000	0.0073	0.0000	0.0000	0.0622	0.0000	0.0000	0.0000
SNB	Est		2.7119	-0.0298	0.0002		1.6553	-0.0381	0.0025	3.2896	2.2350	-1.0546	3.0247	1.8330	-1.1917
	SE		0.1038	0.0075	0.0007		0.0825	0.0064	0.0006	0.1551	0.3172	0.2941	0.1312	0.2678	0.2494
	PT		0.0000	0.0001	0.7179		0.0000	0.0000	0.0000	0.0000	0.0000	0.0003	0.0000	0.0000	0.0000
YLR	Est		1.4336	-0.0015	0.0009		1.0786	-0.0089	0.0007	1.7330	1.7472	0.0143	1.4271	1.1573	-0.2698
	SE		0.1081	0.0065	0.0006		0.0508	0.0031	0.0003	0.2099	0.1615	0.2182	0.0987	0.0762	0.1030
	PT		0.0000	0.8185	0.1427		0.0000	0.0041	0.0256	0.0000	0.0000	0.9479	0.0000	0.0000	0.0088
DTR	Est		3.2249	-0.0650	0.0006		1.8497	-0.0165	-0.0003	3.4950	2.2200	-1.2750	1.9104	1.4115	-0.4989
	SE		0.1722	0.0322	0.0021	.	0.1054	0.0214	0.0014	0.2602	0.3060	0.3160	0.1670	0.1976	0.2069
	PT		0.0000	0.0438	0.7575	.	0.0000	0.4387	0.8021	0.0000	0.0000	0.0001	0.0000	0.0000	0.0159

YLD Grain yield; *MLD* Mildew; *BNR* Brown rust; *STB* Septoria tritici blotch; *SNB* Septoria nodorum blotch; *YLR* Yellow rust; *DTR* Tan spot;

Est estimate of regression coefficient; *SE* standard error; *PT* p-value of t.

Table S1 b) Estimates of regression coefficients for trends of variety means

		Trends of variety means											
		Regression coefficients I1			Regression coefficients I2			Levels I1			Levels I2		
		μ	α	β	μ	α	β	1983	2017	Diff I1	1983	2017	Diff I2
YLD	Est	86.1211	0.5750	-0.0114	98.6967	0.5803	-0.0211	73.0615	92.6130	19.5515	82.7217	102.4523	19.7306
	SE	0.2378	0.0166	0.0016	0.2577	0.0180	0.0018	0.4745	0.4239	0.5638	0.5143	0.4594	0.6111
	PT	0.0000	0.0000	0.0000	0.0000	0.0000	0.0000	0.0000	0.0000	0.0000	0.0000	0.0000	0.0000
MLD	Est	2.3590	-0.0340	0.0003	1.2484	-0.0289	0.0018	3.0266	1.8719	-1.1547	2.2461	1.2626	-0.9835
	SE	0.0324	0.0023	0.0002	0.0132	0.0009	0.0001	0.0647	0.0578	0.0769	0.0263	0.0235	0.0313
	PT	0.0000	0.0000	0.1650	0.0000	0.0000	0.0000	0.0000	0.0000	0.0000	0.0000	0.0000	0.0000
BNR	Est	2.4135	-0.0159	0.0011	1.1514	-0.0267	0.0019	2.9933	2.4541	-0.5392	2.1432	1.2343	-0.9089
	SE	0.0416	0.0029	0.0003	0.0142	0.0010	0.0001	0.0831	0.0742	0.0987	0.0284	0.0254	0.0338
	PT	0.0000	0.0000	0.0002	0.0000	0.0000	0.0000	0.0000	0.0000	0.0000	0.0000	0.0000	0.0000
STB	Est	3.7667	-0.0187	-0.0004	2.0429	-0.0364	0.0017	3.9592	3.3226	-0.6366	3.1567	1.9200	-1.2367
	SE	0.0263	0.0018	0.0002	0.0167	0.0012	0.0001	0.0524	0.0468	0.0623	0.0333	0.0297	0.0395
	PT	0.0000	0.0000	0.0170	0.0000	0.0000	0.0000	0.0000	0.0000	0.0000	0.0000	0.0000	0.0000
SNB	Est	2.5619	-0.0258	0.0011	1.5845	-0.0284	0.0022	3.3049	2.4278	-0.8771	2.7071	1.7418	-0.9653
	SE	0.0226	0.0016	0.0002	0.0170	0.0012	0.0001	0.0449	0.0428	0.0546	0.0338	0.0322	0.0411
	PT	0.0000	0.0000	0.0000	0.0000	0.0000	0.0000	0.0000	0.0000	0.0000	0.0000	0.0000	0.0000
YLR	Est	1.5090	0.0036	0.0013	1.0643	-0.0049	0.0008	1.8326	1.9559	0.1233	1.3716	1.2055	-0.1662
	SE	0.0343	0.0023	0.0002	0.0105	0.0007	0.0001	0.0668	0.0596	0.0793	0.0204	0.0182	0.0242
	PT	0.0000	0.1200	0.0000	0.0000	0.0000	0.0000	0.0000	0.0000	0.1200	0.0000	0.0000	0.0000
DTR	Est	2.8819	-0.0122	-0.0021	1.6067	-0.0062	-0.0004	2.8971	2.0657	-0.8315	1.6254	1.3881	-0.2373
	SE	0.0287	0.0053	0.0004	0.0134	0.0025	0.0002	0.0390	0.0539	0.0661	0.0183	0.0252	0.0309
	PT	0.0000	0.0219	0.0000	0.0000	0.0126	0.0487	0.0000	0.0000	0.0000	0.0000	0.0000	0.0000

YLD Grain yield; *MLD* Mildew; *BNR* Brown rust; *STB* Septoria tritici blotch; *SNB* Septoria nodorum blotch; *YLR* Yellow rust; *DTR* Tan spot;

Est estimate of regression coefficient; *SE* standard error; *PT* p-value of t.

Table S1 c) Trends of variety age

			Age trends					
			Regression coefficients I2-I1			Age levels I2-I1		
		ΔP_1	$\Delta P_2/\Delta\mu$	δ_1^1	δ_2^1	year 1	year 20	Diff
YLD	Est	10.0368	9.4704	0.4427	-0.0085	10.4711	14.9317	4.4606
	SE	0.8542	1.0079	0.0802	0.0037	0.833	1.0508	1.2455
	PT	<.0001	<.0001	<.0001	0.0220	<.0001	<.0001	0.0003
MLD	Est		-0.5818	-0.0594	0.0002	-0.6410	-1.6814	-1.0404
	SE		0.1076	0.0101	0.0004	0.1047	0.1282	0.0912
	PT		0.0000	0.0000	0.6212	0.0000	0.0000	0.0000
BNR	Est		-0.3772	-0.1841	0.0048	-0.5565	-2.1546	-1.5981
	SE		0.1530	0.0189	0.0009	0.1459	0.1882	0.1544
	PT		0.0137	0.0000	0.0000	0.0001	0.0000	0.0000
STB	Est		-1.2527	-0.0487	0.0009	-1.3005	-1.8766	-0.5760
	SE		0.1040	0.0091	0.0004	0.1014	0.1182	0.0805
	PT		0.0000	0.0000	0.0258	0.0000	0.0000	0.0000
SNB	Est		-0.7234	-0.0154	0.0002	-0.7385	-0.9425	-0.2040
	SE		0.0834	0.0094	0.0004	0.0798	0.0927	0.0685
	PT		0.0000	0.1008	0.5940	0.0000	0.0000	0.0029
YLR	Est		-0.1762	-0.0732	0.0016	-0.2478	-1.0014	-0.7535
	SE		0.1316	0.0210	0.0009	0.1201	0.1623	0.1589
	PT		0.1808	0.0005	0.0853	0.0391	0.0000	0.0000
DTR	Est		-1.1350	-0.0224	0.0008	-1.1566	-1.2468	-0.0902
	SE		0.1483	0.0111	0.0005	0.1452	0.1586	0.0938
	PT		0.0000	0.0436	0.0776	0.0000	0.0000	0.3363

YLD Grain yield; *MLD* Mildew; *BNR* Brown rust; *STB* Septoria tritici blotch; *SNB* Septoria nodorum blotch; *YLR* Yellow rust; *DTR* Tan spot;

Est estimate of regression coefficient; *SE* standard error; *PT* p-value of t.
