# Supplementary material for: Breeding progress of disease resistance and impact of disease severity under natural infections in winter wheat variety trials
Source: Theor Appl Genet. 2021 Mar 13;134(5):1281–302. doi: 10.1007/s00122-020-03728-4 (PMC8081715; doi:10.1007/s00122-020-03728-4)
Supplement: Supplementary file 3 — Supplementary file3 (PDF 28 kb) [file 122_2020_3728_MOESM3_ESM.pdf]

Supplementary Material SM3

Table S2 Description of of landmark varieties

					Variety means								
			**Trial year**		**(dt ha^{-1})**		**Variety disease susceptibility I1**						
**Variety**	**Type**	**N**	**First**	**Last**	**YLD I1**	**YLD I2**	**MLD**	**BNR**	**STB**	**SNB**	**YLR**	**DTR**
Kanzler	susceptible	471	1977	1992	68.2	82.0	5.6	4.7	4.2	3.7	4.6	-
Bussard	susceptible	1298	1987	2012	71.8	86.8	3.0	4.2	4.7	2.6	1.6	3.1
Ritmo	susceptible	485	1990	2002	79.7	99.1	3.4	4.1	4.6	3.2	2.2	3.4
Drifter	susceptible	466	1996	2006	81.7	99.0	2.4	2.8	4.9	2.5	1.5	3.4
JB Asano	susceptible	346	2005	2014	86.9	104.8	2.2	3.3	5.0	3.0	4.9	3.1
Greif	resistant	51	1986	1988	75.8	82.7	1.5	2.9	4.0	2.3	1.2	-
Batis	resistant	1031	1991	2011	84.1	96.4	2.8	2.3	3.7	2.7	1.4	2.9
Cardos	resistant	119	1995	1999	81.6	93.0	2.0	1.5	3.4	3.1	1.4	3.2
Tommi	resistant	492	1999	2010	86.6	99.9	1.9	4.0	3.6	2.3	1.3	3.1
Julius	resistant	543	2005	2017	88.2	101.3	2.6	3.0	3.3	2.7	1.9	2.6
			**Mean**		**80.5**	**94.5**	**2.8**	**3.3**	**4.1**	**2.8**	**2.2**	**3.1**

YLD Grain yield; *MLD* Milldew; *BNR* Brown rust; *STB* Septoria tritici blotch; *SNB* Septoria nodorum blotch; *YLR* Yellow rust; *DTR* Tan spot; *N* Number of observations; *I1* Intensity 1; *I2* Intensity 2;
