# Supplementary material for: Breeding progress of disease resistance and impact of disease severity under natural infections in winter wheat variety trials
Source: Theor Appl Genet. 2021 Mar 13;134(5):1281–302. doi: 10.1007/s00122-020-03728-4 (PMC8081715; doi:10.1007/s00122-020-03728-4)
Supplement: Supplementary file 4 — Supplementary file4 (PDF 46 kb) [file 122_2020_3728_MOESM4_ESM.pdf]

Supplementary Material SM4

		MLD										
		1	2	2	3	4	5	6	7	8	9	
STB	1	16.5	1.3	1.8	2.0	0.8	0.5	0.3	0.1	0.0	0.0	23.2
	1.5	1.7	0.2	0.2	0.1	0.1	0.0	0.0	0.0	0.0	0.0	2.4
	2	6.7	0.9	0.9	0.6	0.2	0.1	0.0	0.0	0.0	0.0	9.5
	3	14.7	1.5	2.0	2.3	1.0	0.3	0.2	0.0	0.0	0.0	22.1
	4	11.9	1.0	1.6	2.2	1.1	0.5	0.2	0.1	0.0	0.0	18.5
	5	8.9	0.6	0.8	1.3	0.8	0.4	0.1	0.0	0.0	0.0	12.9
	6	5.3	0.3	0.4	0.5	0.2	0.1	0.1	0.0	0.0	0.0	6.9
	7	2.4	0.1	0.2	0.2	0.1	0.1	0.0	0.0	0.0	0.0	3.1
	8	1.0	0.0	0.0	0.1	0.0	0.0	0.0	0.0	0.0	0.0	1.2
	9	0.2	0.0	0.0	0.0	0.0	0.0	0.0	0.0	0.0	0.0	0.3
		69.3	5.8	7.8	9.3	4.5	1.9	0.9	0.3	0.1	0.0	100.0

		STB										
		1	2	2	3	4	5	6	7	8	9	
YLR	1	18.5	2.0	7.9	18.1	15.1	10.9	5.8	2.7	1.0	0.2	82.3
	2	1.1	0.1	0.4	0.8	0.5	0.2	0.2	0.1	0.0	0.0	3.5
	2	1.3	0.1	0.5	1.1	0.8	0.3	0.2	0.1	0.0	0.0	4.4
	3	1.0	0.1	0.4	1.2	1.0	0.5	0.2	0.1	0.0	0.0	4.6
	4	0.5	0.0	0.1	0.5	0.6	0.4	0.2	0.1	0.0	0.0	2.4
	5	0.2	0.0	0.1	0.2	0.3	0.3	0.1	0.0	0.0	0.0	1.3
	6	0.2	0.0	0.0	0.1	0.2	0.2	0.1	0.0	0.0	0.0	0.8
	7	0.1	0.0	0.0	0.1	0.1	0.1	0.1	0.0	0.0	0.0	0.6
	8	0.1	0.0	0.0	0.0	0.0	0.0	0.1	0.0	0.0	0.0	0.3
	9	0.0	0.0	0.0	0.0	0.0	0.0	0.0	0.0	0.0	0.0	0.1
		23.2	2.4	9.5	22.1	18.5	12.9	6.9	3.1	1.2	0.3	100.0

		BNR										
		1	2	2	3	4	5	6	7	8	9	
STB	1	12.2	1.5	1.8	2.6	1.6	1.1	1.0	0.6	0.4	0.3	23.2
	1.5	1.3	0.3	0.2	0.2	0.1	0.1	0.0	0.0	0.0	0.0	2.4
	2	4.9	1.0	1.3	1.1	0.4	0.3	0.2	0.2	0.1	0.1	9.5
	3	12.0	1.6	2.4	2.6	1.3	0.9	0.5	0.3	0.3	0.1	22.1
	4	9.5	1.2	2.0	2.4	1.5	0.9	0.4	0.3	0.2	0.1	18.5
	5	6.9	0.8	1.1	1.6	1.0	0.6	0.4	0.2	0.1	0.2	12.9
	6	3.9	0.4	0.4	0.7	0.5	0.4	0.3	0.2	0.1	0.1	6.9
	7	2.0	0.1	0.2	0.3	0.2	0.1	0.1	0.1	0.0	0.0	3.1
	8	0.9	0.0	0.1	0.0	0.0	0.0	0.0	0.0	0.0	0.0	1.2
	9	0.2	0.0	0.0	0.0	0.0	0.0	0.0	0.0	0.0	0.0	0.3
		53.8	7.0	9.5	11.5	6.5	4.5	3.1	2.0	1.3	0.8	100.0

		MLD										
		1	2	2	3	4	5	6	7	8	9	
SNB	1	64.2	5.0	6.6	8.4	3.9	1.6	0.8	0.3	0.1	0.0	90.9
	2	0.6	0.1	0.1	0.1	0.1	0.0	0.0	0.0	0.0	0.0	1.0
	2	1.8	0.4	0.5	0.4	0.2	0.1	0.1	0.0	0.0	0.0	3.5
	3	1.8	0.2	0.3	0.3	0.2	0.2	0.1	0.0	0.0	0.0	3.1
	4	0.6	0.1	0.1	0.1	0.1	0.0	0.0	0.0	0.0	0.0	1.0
	5	0.3	0.0	0.0	0.0	0.0	0.0	0.0	0.0	0.0	0.0	0.4
	6	0.1	0.0	0.0	0.0	0.0	0.0	0.0	0.0	0.0	0.0	0.1
	7	0.0	0.0	0.0	0.0	0.0	0.0	0.0	0.0	0.0	0.0	0.0
		69.3	5.8	7.8	9.3	4.5	1.9	0.9	0.3	0.1	0.0	100.0

		BNR										
		1	2	2	3	4	5	6	7	8	9	
MLD	1	40.2	4.7	5.4	7.2	4.0	2.8	1.9	1.3	1.0	0.7	69.3
	1.5	2.9	0.6	0.6	0.7	0.3	0.3	0.2	0.1	0.1	0.0	5.8
	2	3.3	0.5	1.4	1.2	0.6	0.4	0.3	0.1	0.0	0.0	7.8
	3	4.2	0.7	1.3	1.3	0.8	0.5	0.3	0.2	0.1	0.0	9.3
	4	2.0	0.3	0.4	0.7	0.4	0.2	0.2	0.1	0.0	0.0	4.5
	5	0.8	0.2	0.2	0.3	0.2	0.2	0.1	0.1	0.0	0.0	1.9
	6	0.3	0.1	0.1	0.2	0.1	0.1	0.0	0.0	0.0	0.0	0.9
	7	0.1	0.0	0.0	0.1	0.0	0.0	0.0	0.0	0.0	0.0	0.3
	8	0.0	0.0	0.0	0.0	0.0	0.0	0.0	0.0	0.0	0.0	0.1
	9	0.0	0.0	0.0	0.0	0.0	0.0	0.0	0.0	0.0	0.0	0.0
		53.8	7.0	9.5	11.5	6.5	4.5	3.1	2.0	1.3	0.8	100.0

		SNB								
		1	2	2	3	4	5	6	7	
YLR	1	75.3	0.9	2.9	2.4	0.5	0.1	0.0	0.0	82.3
	2	3.3	0.0	0.1	0.1	0.0	0.0	0.0	0.0	3.5
	2	3.8	0.0	0.2	0.2	0.1	0.1	0.0	0.0	4.4
	3	4.1	0.0	0.1	0.2	0.1	0.0	0.0	0.0	4.6
	4	2.0	0.0	0.1	0.1	0.1	0.1	0.0	0.0	2.4
	5	1.1	0.0	0.0	0.1	0.1	0.0	0.0	0.0	1.3
	6	0.6	0.0	0.0	0.0	0.0	0.0	0.0	0.0	0.8
	7	0.4	0.0	0.0	0.0	0.0	0.0	0.0	0.0	0.6
	8	0.2	0.0	0.0	0.0	0.0	0.0	0.0	0.0	0.3
	9	0.1	0.0	0.0	0.0	0.0	0.0	0.0	0.0	0.1
		90.9	1.0	3.5	3.1	1.0	0.4	0.1	0.0	100.0

Fig. S1 Two-dimensional frequency table with marginal distributions of disease severity scores as percent of total number of observations. (n= 23,083) from data 2005 - 2019.

MLD Mildew; *BNR* Brown rust; *STB* Septoria tritici blotch; *SNB* Septoria nodorum blotch; *YLR* Yellow rust;
