# Supplementary material for: Breeding progress of disease resistance and impact of disease severity under natural infections in winter wheat variety trials
Source: Theor Appl Genet. 2021 Mar 13;134(5):1281–302. doi: 10.1007/s00122-020-03728-4 (PMC8081715; doi:10.1007/s00122-020-03728-4)
Supplement: Supplementary file 5 — Supplementary file5 (PDF 203 kb) [file 122_2020_3728_MOESM5_ESM.pdf]

Supplementary Material SM5

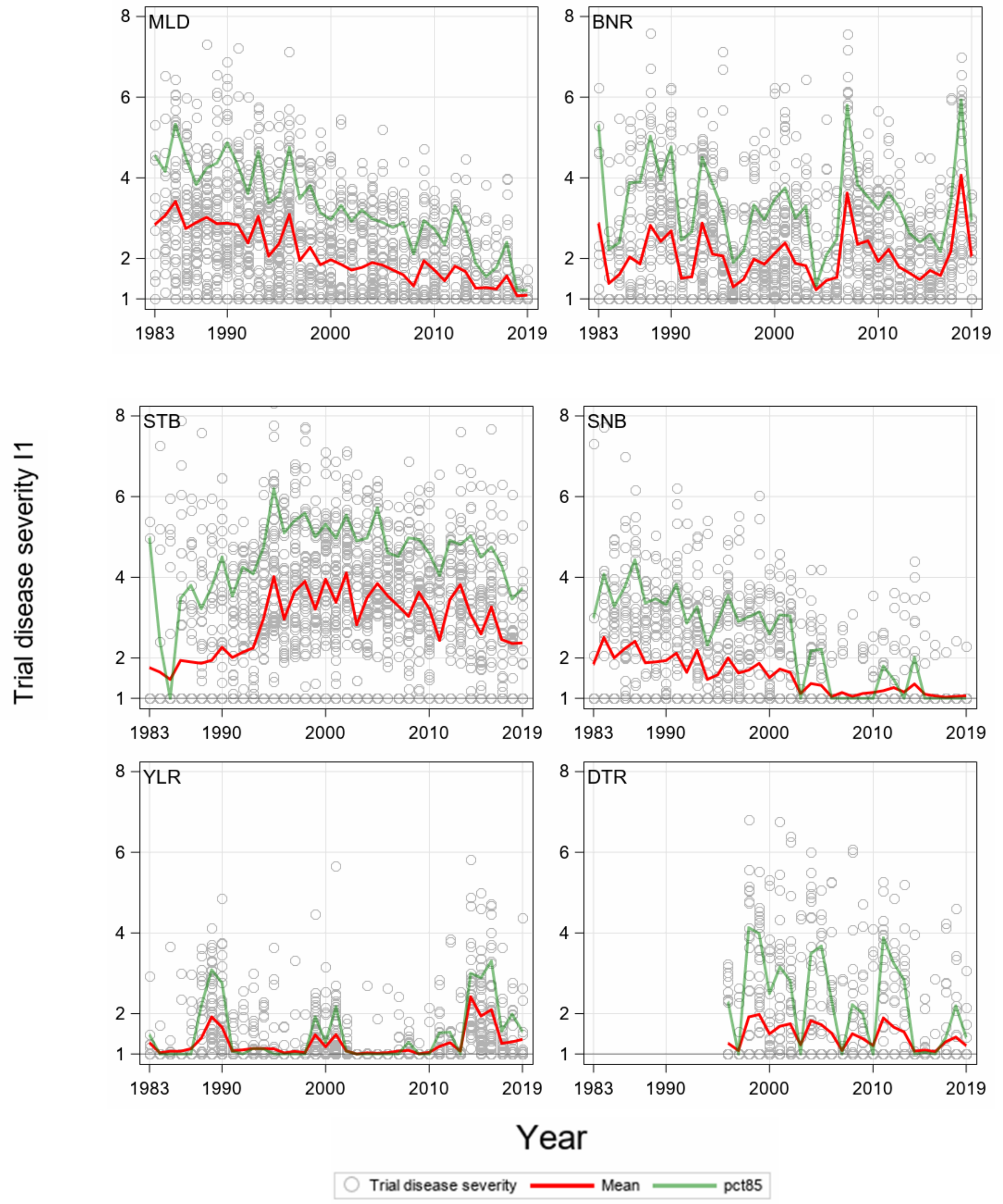

Fig. S2 Trial disease severity 1983 (1996 for DTR)- 2019
Trial disease severity: Average of disease severity scores over all varieties within a trial; *Mean*: Mean of trial disease severity over trials within year; *pctl85*: 85th percentile of trial disease severity within year.
MLD Mildew; *BNR* Brown rust; *STB* Septoria tritici blotch*; SNB* Septoria nodorum blotch; *YLR* Yellow rust; *DTR* Tan spot; *I1* Intensity 1; *I2* Intensity 2;
