# Supplementary material for: Breeding progress of disease resistance and impact of disease severity under natural infections in winter wheat variety trials
Source: Theor Appl Genet. 2021 Mar 13;134(5):1281–302. doi: 10.1007/s00122-020-03728-4 (PMC8081715; doi:10.1007/s00122-020-03728-4)
Supplement: Supplementary file 6 — Supplementary file6 (PDF 92 kb) [file 122_2020_3728_MOESM6_ESM.pdf]

Supplementary Material SM6

MLD		TSv						
		1	1.5	2	3	4	5	
VSc	1	1.0	0.2	0.1	0.1	0.0	0.0	**1.4**
	1.5	18.9	4.0	3.8	2.7	0.7	0.0	**30.3**
	2	25.9	5.7	6.5	5.0	1.4	0.2	**44.6**
	3	12.6	2.8	3.4	2.8	0.8	0.1	**22.5**
	4	0.7	0.1	0.2	0.1	0.1	0.0	**1.2**
		**59.1**	**12.8**	**14.1**	**10.7**	**3.0**	**0.3**	**100.0**

BNR		TSv									
		1.0	1.5	2.0	3.0	4.0	5.0	6.0	7.0	8.0	
VSc	1	0.3	0.1	0.2	0.1	0.0	0.0	0.0	0.0	0.0	**0.8**
	1.5	8.8	2.9	4.0	3.3	1.5	0.4	0.3	0.0	0.0	**21.2**
	2	13.6	4.0	5.4	4.6	2.5	0.7	0.5	0.1	0.0	**31.4**
	3	10.4	3.3	5.4	5.0	3.0	0.9	1.0	0.2	0.1	**29.2**
	4	5.5	1.8	2.7	2.7	1.7	0.5	0.5	0.1	0.0	**15.5**
	5	0.5	0.2	0.3	0.4	0.2	0.0	0.1	0.0	0.0	**1.8**
		**39.2**	**12.2**	**18.1**	**16.0**	**8.9**	**2.6**	**2.4**	**0.5**	**0.2**	**100.0**

STB		TSv									
		1.0	1.5	2.0	3.0	4.0	5.0	6.0	7.0	8.0	
VSc	1	0.0	0.0	0.0	0.0	0.0	0.0	0.0	0.0	0.0	**0.0**
	1.5	0.0	0.0	0.0	0.0	0.0	0.0	0.0	0.0	0.0	**0.0**
	2	0.4	0.0	0.1	0.3	0.2	0.1	0.0	0.0	0.0	**1.1**
	3	11.0	1.1	5.4	10.9	10.4	5.2	1.8	0.0	0.0	**45.8**
	4	8.9	0.7	4.0	10.8	11.9	6.6	2.5	0.3	0.1	**45.8**
	5	1.1	0.1	0.5	1.6	2.0	1.0	0.4	0.3	0.1	**7.1**
	6	0.0	0.0	0.0	0.0	0.0	0.0	0.0	0.0	0.0	**0.2**
		**21.5**	**1.9**	**10.0**	**23.6**	**24.5**	**12.9**	**4.7**	**0.6**	**0.3**	**100.0**

SNB		TSv					
		1.0	1.5	2.0	3.0	4.0	
VSc	1	3.7	0.0	0.0	0.0	0.0	**0.0**
	1.5	3.0	0.1	0.2	0.0	0.0	**3.2**
	2	43.6	0.9	2.7	1.1	0.3	**48.7**
	3	38.4	0.4	2.3	1.0	0.8	**42.8**
	4	1.4	0.0	0.0	0.0	0.0	**1.5**
		**86.3**	**1.3**	**5.2**	**2.3**	**1.1**	**96.3**

YLR		TSv						
		1.0	1.5	2.0	3.0	4.0	5.0	6	
VSc	1	16.0	0.9	0.3	0.1	0.1	0.0	0.0	**17.4**
	1.5	33.2	4.7	3.0	1.6	0.7	0.3	0.0	**43.5**
	2	16.2	4.2	4.1	2.5	0.8	0.5	0.1	**28.3**
	3	3.0	0.7	0.8	0.5	0.1	0.1	0.0	**5.3**
	4	2.0	0.5	0.5	0.3	0.1	0.1	0.0	**3.4**
	5	1.6	0.2	0.2	0.1	0.0	0.0	0.0	**2.1**
		**72.0**	**11.2**	**8.8**	**5.1**	**1.8**	**0.9**	**0.2**	**100.0**

Fig. S3 Two-dimensional frequency table with marginal distributions of variety disease susceptibility (VSc) and trial disease severity (TSv) as percent of total number of observations. (n= 23,083) from data 2005 – 2019

MLD Mildew; *BNR* Brown rust; *STB* Septoria tritici blotch; *SNB* Septoria nodorum blotch; YLR Yellow rust;
