# Supplementary material for: Breeding progress of disease resistance and impact of disease severity under natural infections in winter wheat variety trials
Source: Theor Appl Genet. 2021 Mar 13;134(5):1281–302. doi: 10.1007/s00122-020-03728-4 (PMC8081715; doi:10.1007/s00122-020-03728-4)
Supplement: Supplementary file 7 — Supplementary file7 (PDF 268 kb) [file 122_2020_3728_MOESM7_ESM.pdf]

Supplementary Material SM7

In this Supplementary Material we compare least square estimates for variety means with back-transformed least square means of logit transformed observations by considering the genotype effect G_i in Eq. (1a) as fixed.

Least square means of varieties

a) untransformed b) logit back-transformed

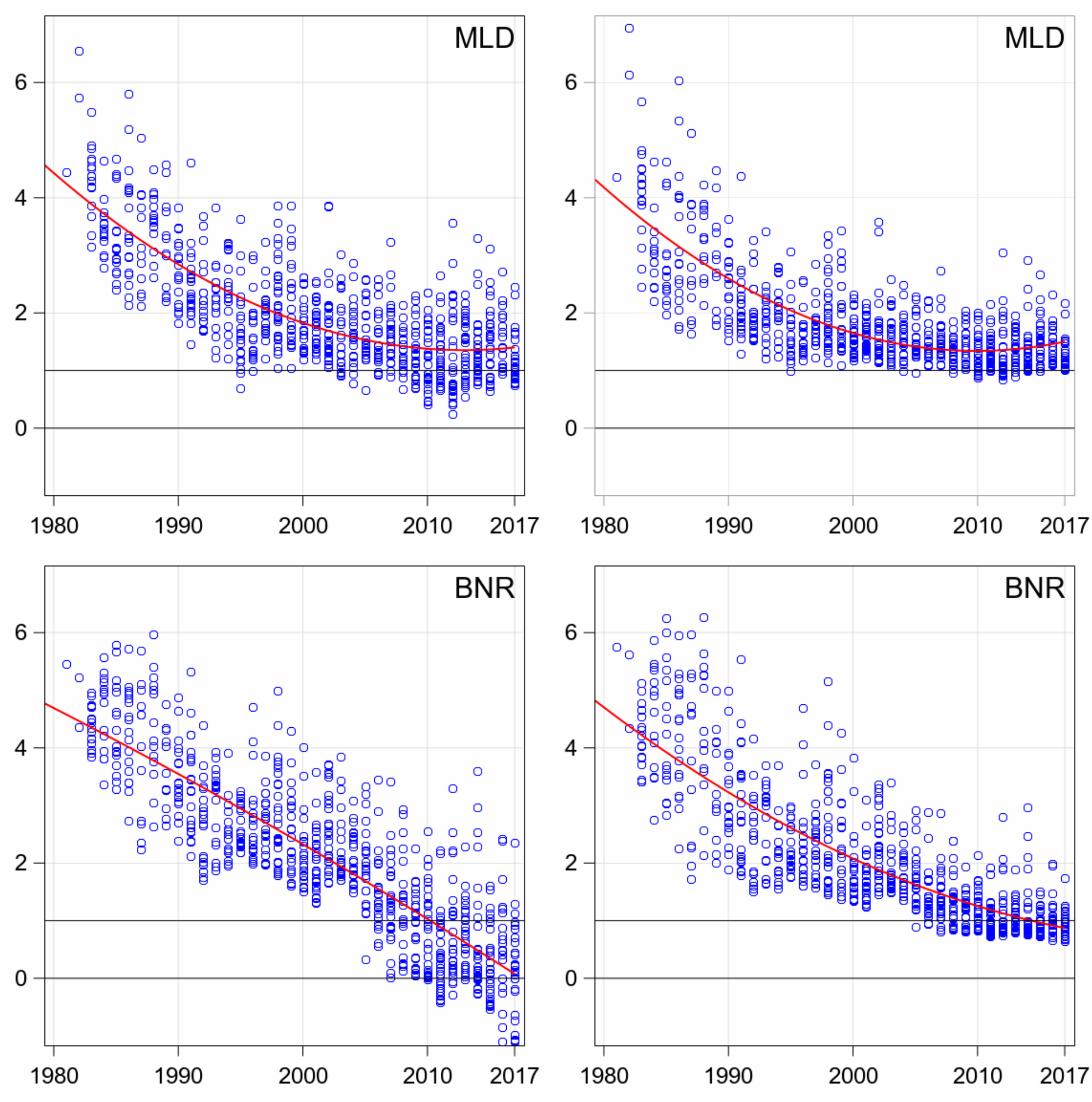

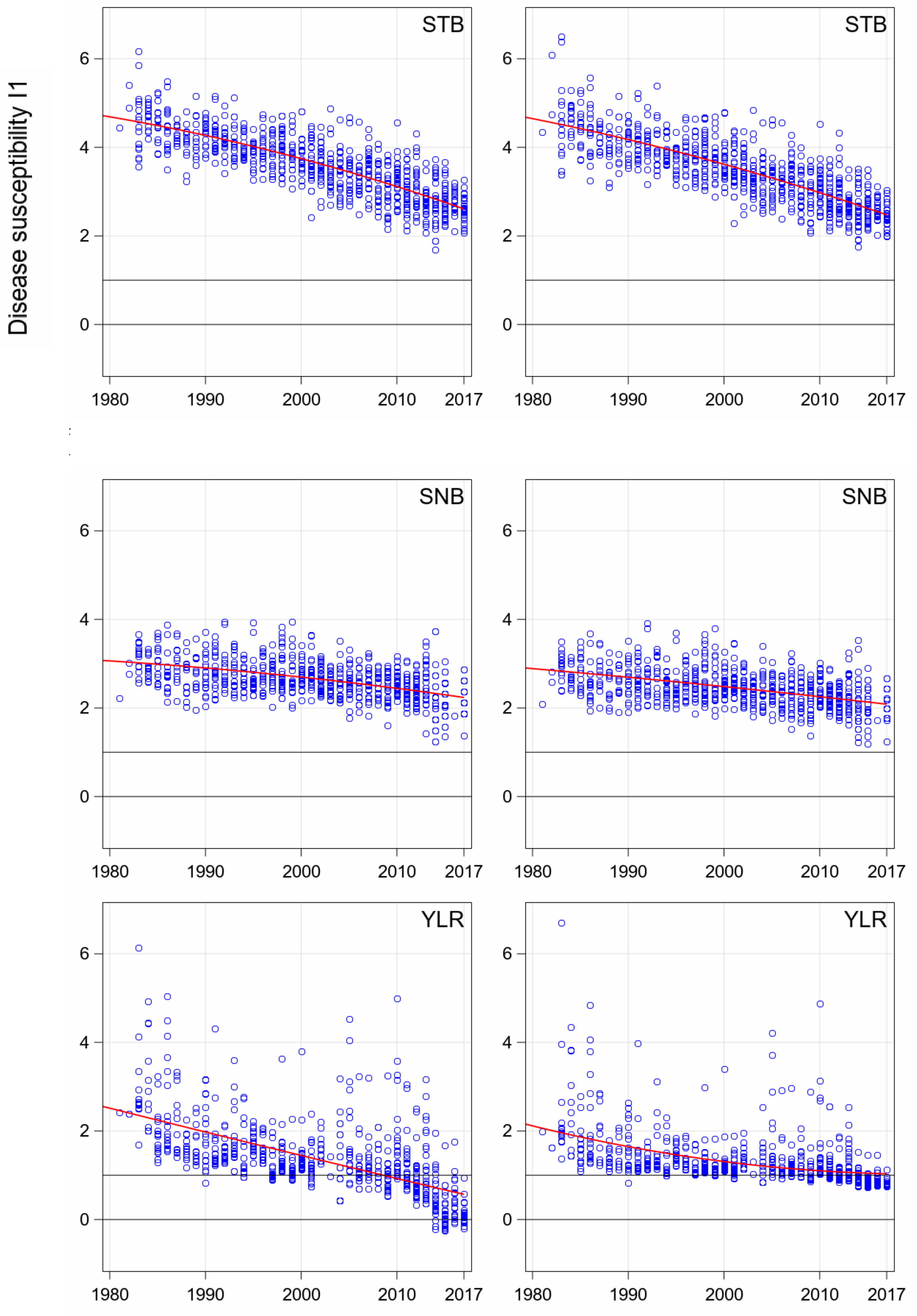
Disease susceptibility I1
STB
STB
SNB
SNB
YLR
YLR
6
4
2
0
1980
1990
2000
2010
2017

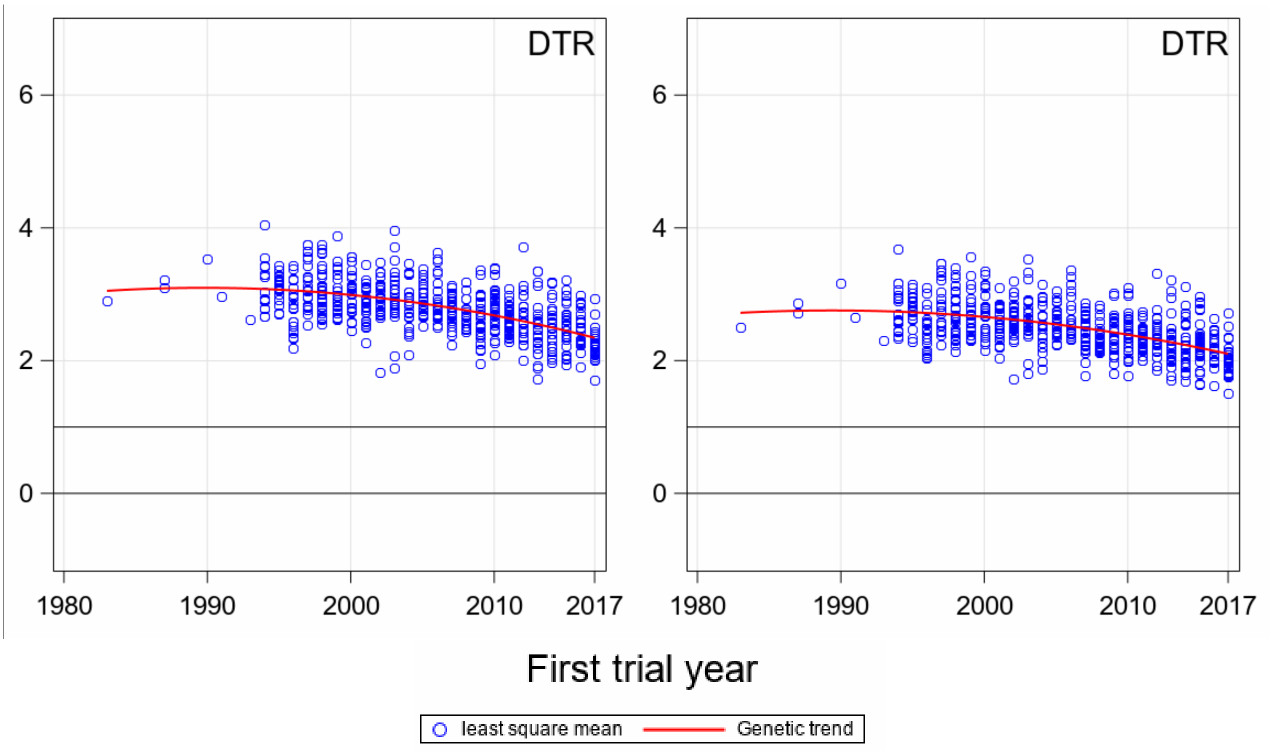

Fig. S4 Variety disease susceptibility (VSc) I1plotted against first trial year. a) left column: least square means.
b) right column: back transformed least square means estimated from logit transformed observed severity scores
MLD Mildew; *BNR* Brown rust; *STB* Septoria tritici blotch*; SNB* Septoria nodorum blotch;
YLR Yellow rust; DTR Tan spot; *I1* Intensity 1;
