# Supplementary material for: Breeding progress of disease resistance and impact of disease severity under natural infections in winter wheat variety trials
Source: Theor Appl Genet. 2021 Mar 13;134(5):1281–302. doi: 10.1007/s00122-020-03728-4 (PMC8081715; doi:10.1007/s00122-020-03728-4)
Supplement: Supplementary file 8 — Supplementary file8 (PDF 1219 kb) [file 122_2020_3728_MOESM8_ESM.pdf]

Supplementary Material SM8

Why analysing ordinal scores as metric data?

Here we explain why we are confident that the analysis we are performing, i.e. fitting a linear mixed model to the observed scores, is the best option.

(1) We do not claim to be estimating disease severity on a percentage scale. Instead, we simply take the observed scores at face value. The rating scale is essentially a logarithmic scale compared to the underlying percentage scale, with low scores corresponding to small increments on the percentage scale and larger scores corresponding to larger increments. This logarithmic scale is widely used in plant breeding and variety testing for good reasons. It is the most convenient way to assess disease severity. By analysing the scores as is, we are essentially doing the analysis on an approximate logarithmic scale. The main purpose of all analyses is to properly rank the varieties. An absolute and unbiased assessment on a percentage severity scale is unnecessary for this purpose.

(2) It would of course be highly desirable that all disease severity scoring could be done directly on a percentage scale, and one of the authors of the current submissions has strongly argued in this direction (Hartung and Piepho, 2007). It needs to be appreciated, however, that such an assessment requires more effort. For an ordinal rating scale, the rater just needs to assess the appropriate ordinal category. By contrast, for the percentage scale, the rater also has to assess the approximate percentage value within that ordinal category. While such a more refined rating is certainly desirable, it does require somewhat more effort. Considering the large number of characteristics that typically need to be scored in breeding programs and variety testing systems, it is understandable that this extra effort is avoided. Also, there is usually a considerable number of characteristics that do not have an underlying percentage scale. Where a percentage scale is present, modern phenotyping methods may be used more frequently in future to assess these percentages directly. But we are dealing with long-term historical data where the assessment was on an ordinal scale, and our purpose is to make the best use of these very valuable data.

(3) It is sometimes recommended to back transform ordinal scores to the mid-percentages of the nine ordinal categories (Bock et al., 2017). This approach will essentially just replace one ordinal rating scale with another one, as the back transformation will only yield nine different percentage values. These are not expected to yield grossly different results. The main reason for Bock et al. (2017) to recommend this back transformation was their concern with bias when wanting to estimate disease severity unbiasedly on the original percentage scale. But as explained under (1), this is not our intention, so the problem dealt with in Bock et al. (2017) is not an issue in our analysis.

(4) Residual plots for our analyses clearly show that the assumption normality and homogeneity of variance is approximately valid. Of course, these assumptions cannot be perfectly valid, and they don't have, to approximate validity is sufficient for a meaningful analysis by these models.

For all of these reasons, we are convinced that our analysis, despite its obvious imperfections, is the best of all possible alternative methods of analysis.

Residual plots

In Fig. S5 below we show plots of studentized residuals for the models we applied in this study to provide a visual check of their distribution properties as compared to the normal distribution. The plots of the left column show the studentized residuals for yield and diseases of I1 plotted against their predicted values, the middle column depicts the histogram of the

studentized residuals overlaid by the shape of the standard normal density function, in the right column the normal Q-Q-plots are shown.

a)

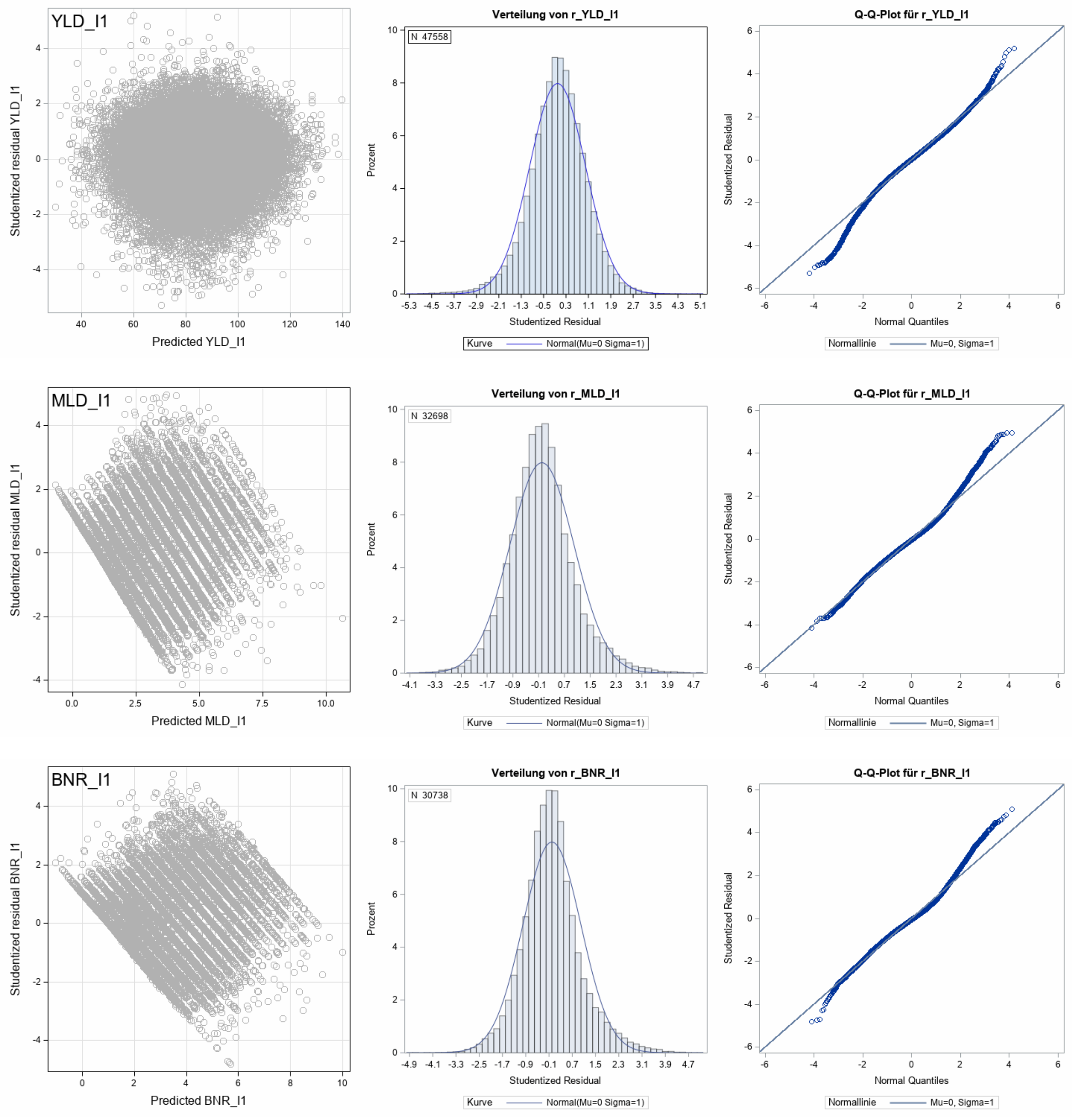

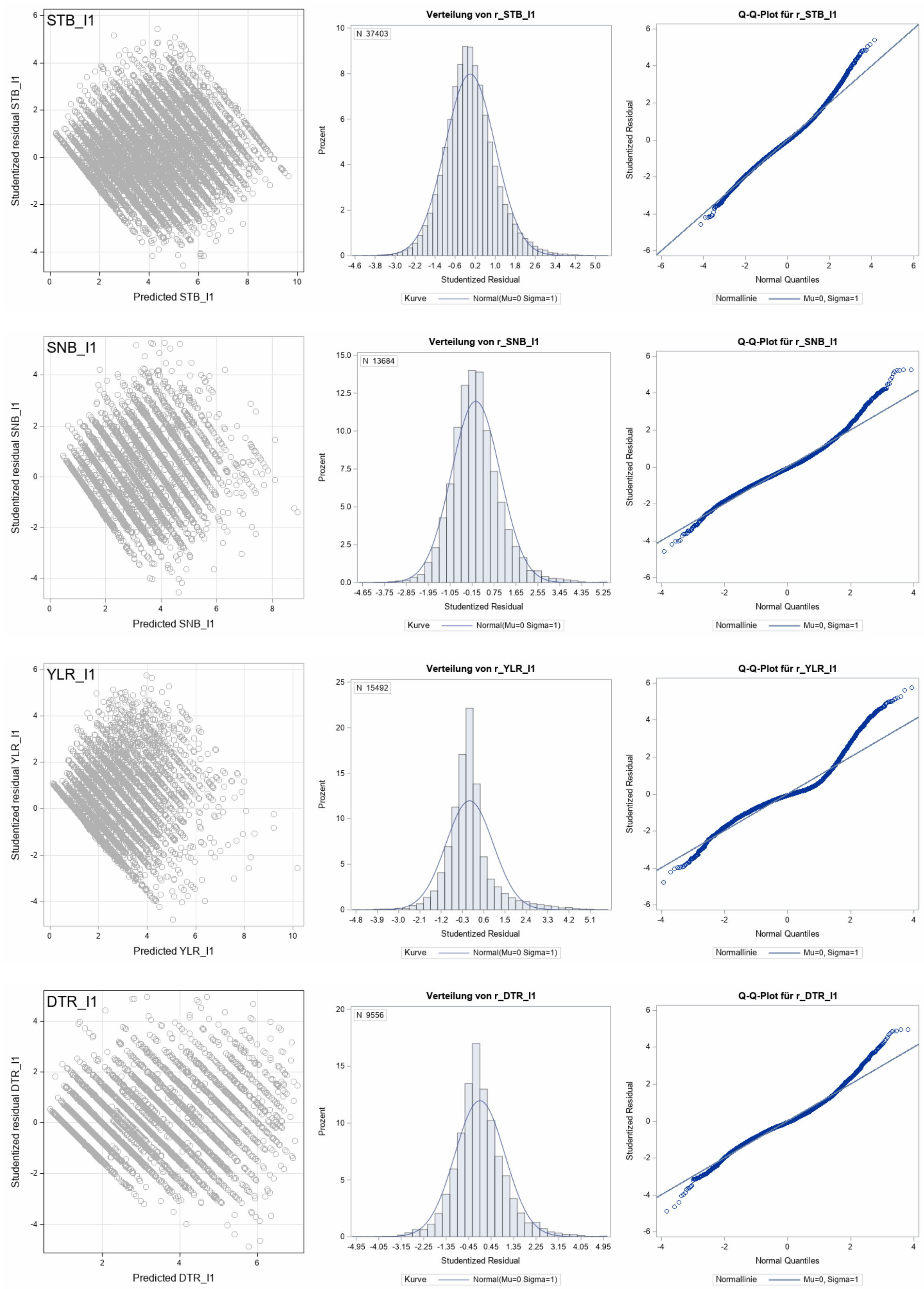
STB_I1
Studentized residual STB_I1
Predicted STB_I1
Verteilung von r_STB_I1
N 37403
Prozent
Studentized Residual
Kurve
Normal(Mu=0 Sigma=1)
Q-Q-Plot für r_STB_I1
Studentized Residual
Normal Quantiles
Normallinie
Mu=0, Sigma=1
SNB_I1
Studentized residual SNB_I1
Predicted SNB_I1
Verteilung von r_SNB_I1
N 13684
Prozent
Studentized Residual
Kurve
Normal(Mu=0 Sigma=1)
Q-Q-Plot für r_SNB_I1
Studentized Residual
Normal Quantiles
Normallinie
Mu=0, Sigma=1
YLR_I1
Studentized residual YLR_I1
Predicted YLR_I1
Verteilung von r_YLR_I1
N 15492
Prozent
Studentized Residual
Kurve
Normal(Mu=0 Sigma=1)
Q-Q-Plot für r_YLR_I1
Studentized Residual
Normal Quantiles
Normallinie
Mu=0, Sigma=1
DTR_I1
Studentized residual DTR_I1
Predicted DTR_I1
Verteilung von r_DTR_I1
N 9556
Prozent
Studentized Residual
Kurve
Normal(Mu=0 Sigma=1)
Q-Q-Plot für r_DTR_I1
Studentized Residual
Normal Quantiles
Normallinie
Mu=0, Sigma=1

b)

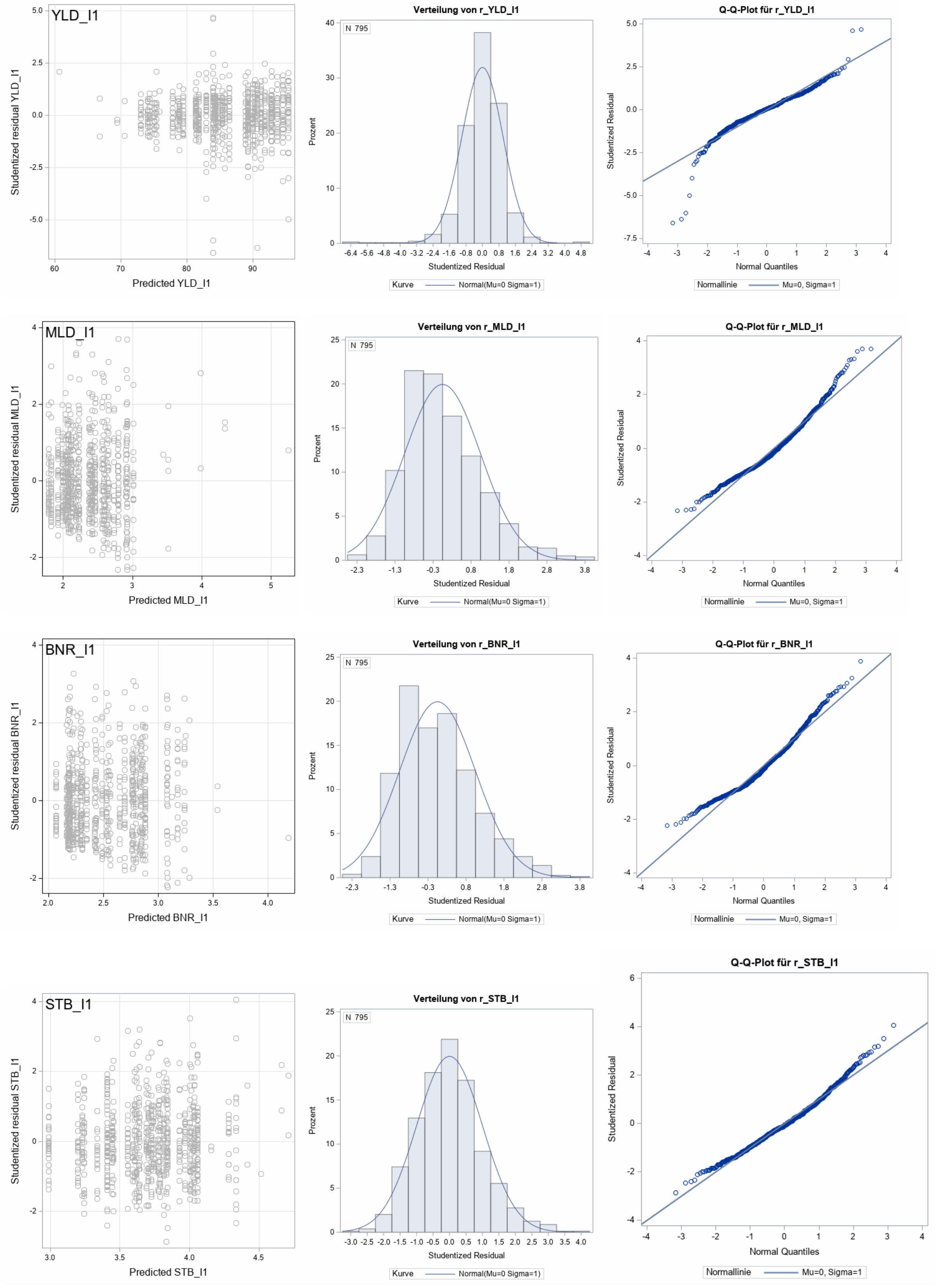

YLD_I1
Studentized residual YLD_I1
Predicted YLD_I1
Verteilung von r_YLD_I1
N 795
Prozent
Studentized Residual
Kurve
Normal(Mu=0 Sigma=1)
Q-Q-Plot für r_YLD_I1
Studentized Residual
Normal Quantiles
Normallinie
Mu=0, Sigma=1
MLD_I1
Studentized residual MLD_I1
Predicted MLD_I1
Verteilung von r_MLD_I1
N 795
Prozent
Studentized Residual
Kurve
Normal(Mu=0 Sigma=1)
Q-Q-Plot für r_MLD_I1
Studentized Residual
Normal Quantiles
Normallinie
Mu=0, Sigma=1
BNR_I1
Studentized residual BNR_I1
Predicted BNR_I1
Verteilung von r_BNR_I1
N 795
Prozent
Studentized Residual
Kurve
Normal(Mu=0 Sigma=1)
Q-Q-Plot für r_BNR_I1
Studentized Residual
Normal Quantiles
Normallinie
Mu=0, Sigma=1
STB_I1
Studentized residual STB_I1
Predicted STB_I1
Verteilung von r_STB_I1
N 795
Prozent
Studentized Residual
Kurve
Normal(Mu=0 Sigma=1)
Q-Q-Plot für r_STB_I1
Studentized Residual
Normal Quantiles
Normallinie
Mu=0, Sigma=1

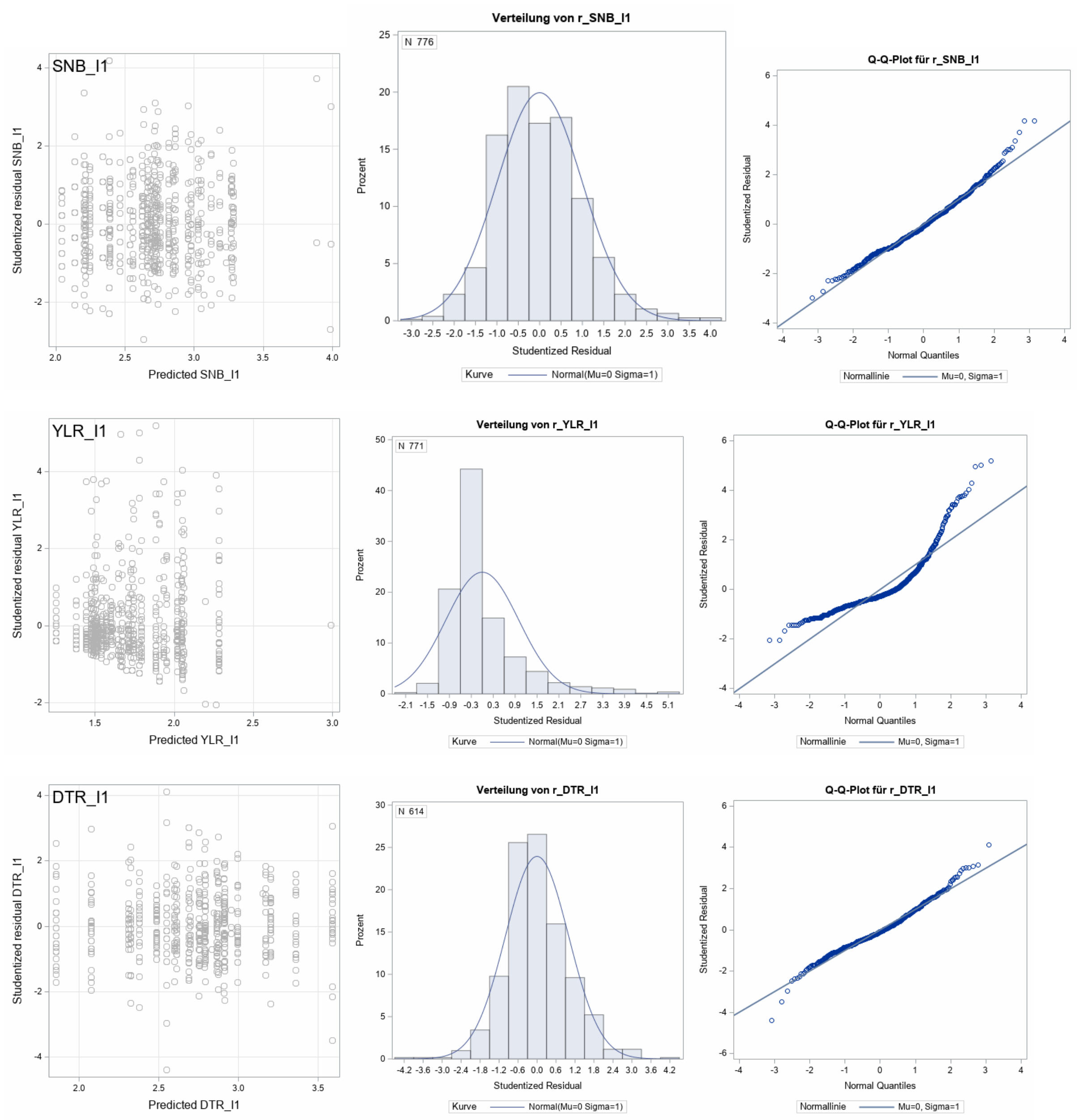

SNB_I1
Studentized residual SNB_I1
Predicted SNB_I1
Verteilung von r_SNB_I1
N 776
Prozent
Studentized Residual
Kurve
Normal(Mu=0 Sigma=1)
Q-Q-Plot für r_SNB_I1
Studentized Residual
Normal Quantiles
Normallinie
Mu=0, Sigma=1
YLR_I1
Studentized residual YLR_I1
Predicted YLR_I1
Verteilung von r_YLR_I1
N 771
Prozent
Studentized Residual
Kurve
Normal(Mu=0 Sigma=1)
Q-Q-Plot für r_YLR_I1
Studentized Residual
Normal Quantiles
Normallinie
Mu=0, Sigma=1
DTR_I1
Studentized residual DTR_I1
Predicted DTR_I1
Verteilung von r_DTR_I1
N 614
Prozent
Studentized Residual
Kurve
Normal(Mu=0 Sigma=1)
Q-Q-Plot für r_DTR_I1
Studentized Residual
Normal Quantiles
Normallinie
Mu=0, Sigma=1

c)

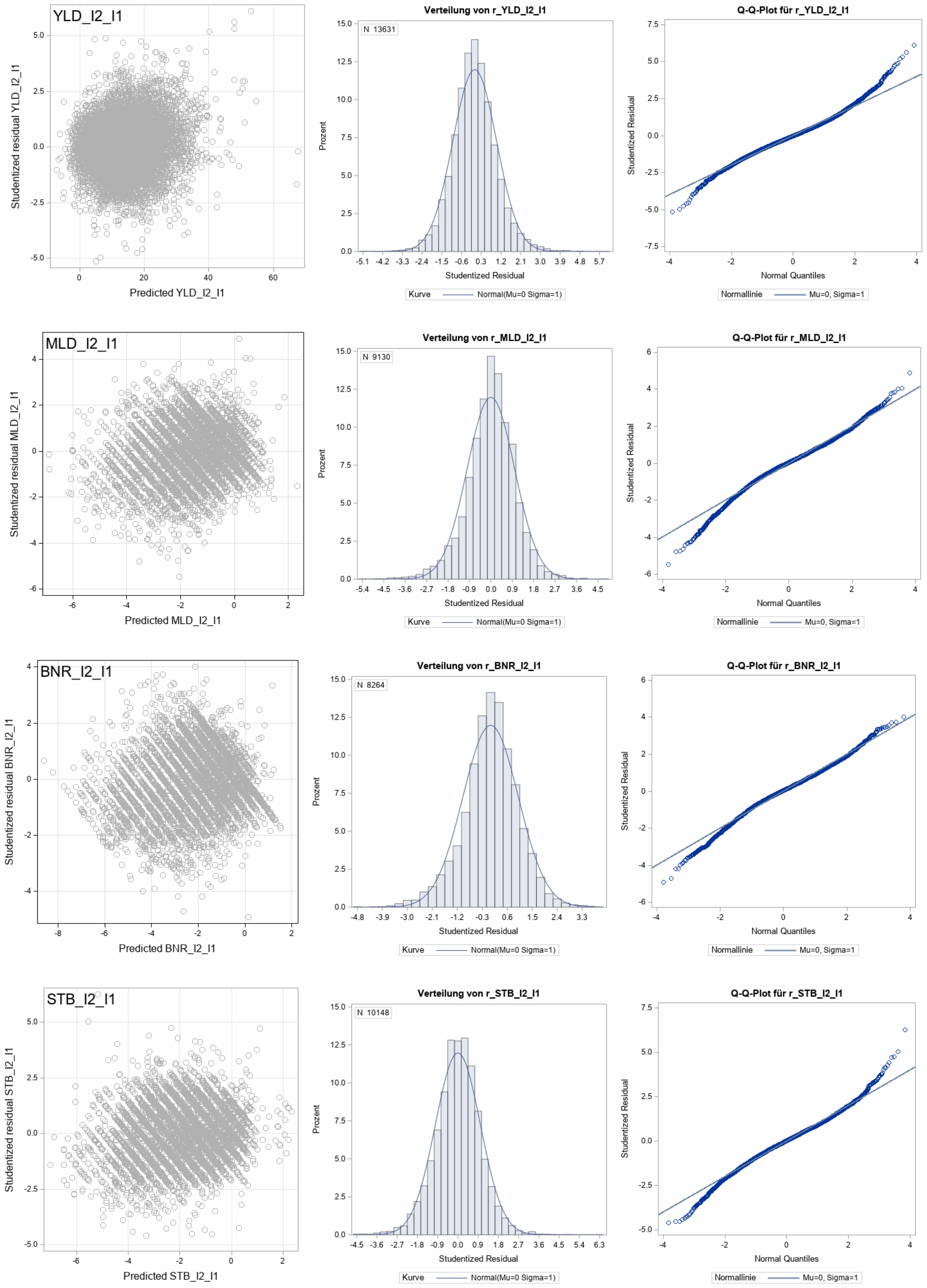
YLD_I2_I1
Studentized residual YLD_I2_I1
Predicted YLD_I2_I1
Verteilung von r_YLD_I2_I1
N 13631
Prozent
Studentized Residual
Kurve
Normal(Mu=0 Sigma=1)
Q-Q-Plot für r_YLD_I2_I1
Studentized Residual
Normal Quantiles
Normallinie
Mu=0, Sigma=1
MLD_I2_I1
Studentized residual MLD_I2_I1
Predicted MLD_I2_I1
Verteilung von r_MLD_I2_I1
N 9130
Prozent
Studentized Residual
Kurve
Normal(Mu=0 Sigma=1)
Q-Q-Plot für r_MLD_I2_I1
Studentized Residual
Normal Quantiles
Normallinie
Mu=0, Sigma=1
BNR_I2_I1
Studentized residual BNR_I2_I1
Predicted BNR_I2_I1
Verteilung von r_BNR_I2_I1
N 8264
Prozent
Studentized Residual
Kurve
Normal(Mu=0 Sigma=1)
Q-Q-Plot für r_BNR_I2_I1
Studentized Residual
Normal Quantiles
Normallinie
Mu=0, Sigma=1
STB_I2_I1
Studentized residual STB_I2_I1
Predicted STB_I2_I1
Verteilung von r_STB_I2_I1
N 10148
Prozent
Studentized Residual
Kurve
Normal(Mu=0 Sigma=1)
Q-Q-Plot für r_STB_I2_I1
Studentized Residual
Normal Quantiles
Normallinie
Mu=0, Sigma=1

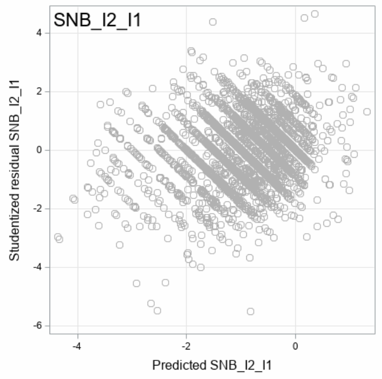
SNB_I2_I1
Studentized residual SNB_I2_I1
Predicted SNB_I2_I1

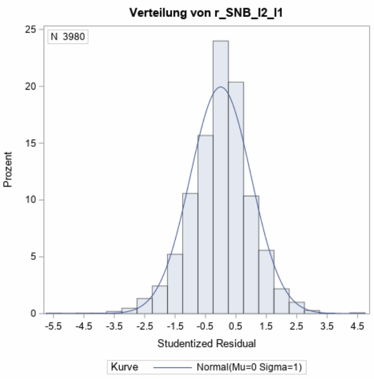
Verteilung von r_SNB_I2_I1
N 3980
Prozent
Studentized Residual
Kurve
Normal(Mu=0 Sigma=1)

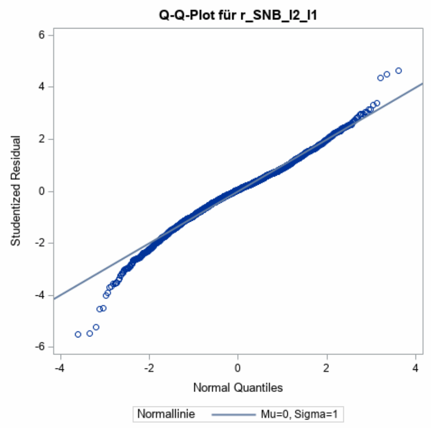
Q-Q-Plot für r_SNB_I2_I1
Studentized Residual
Normal Quantiles
Normallinie
Mu=0, Sigma=1

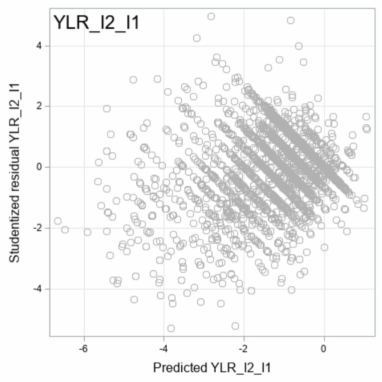
YLR_I2_I1
Studentized residual YLR_I2_I1
Predicted YLR_I2_I1

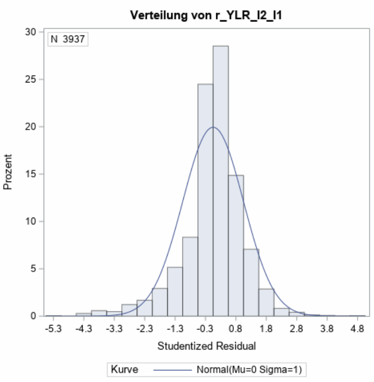
Verteilung von r_YLR_I2_I1
N 3937
Prozent
Studentized Residual
Kurve
Normal(Mu=0 Sigma=1)

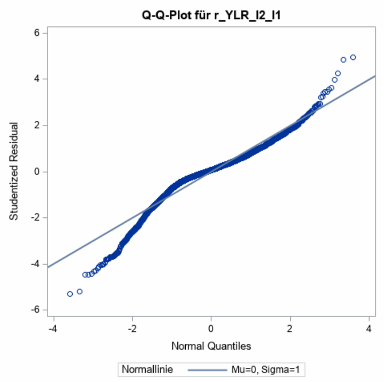
Q-Q-Plot für r_YLR_I2_I1
Studentized Residual
Normal Quantiles
Normallinie
Mu=0, Sigma=1

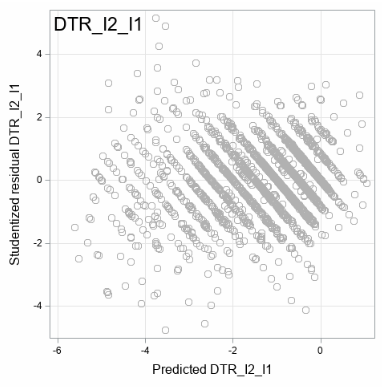
DTR_I2_I1
Studentized residual DTR_I2_I1
Predicted DTR_I2_I1

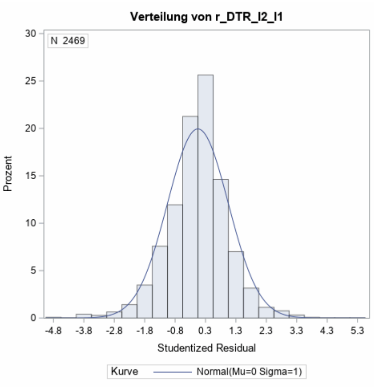
Verteilung von r_DTR_I2_I1
N 2469
Prozent
Studentized Residual
Kurve
Normal(Mu=0 Sigma=1)

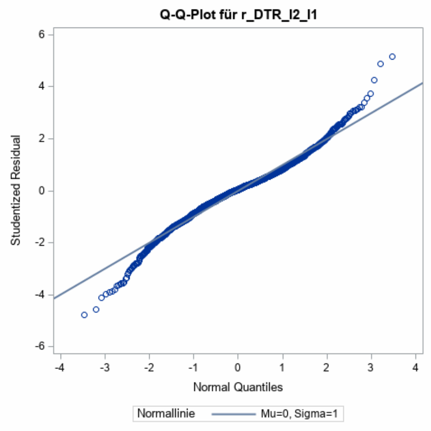
Q-Q-Plot für r_DTR_I2_I1
Studentized Residual
Normal Quantiles
Normallinie
Mu=0, Sigma=1

d)

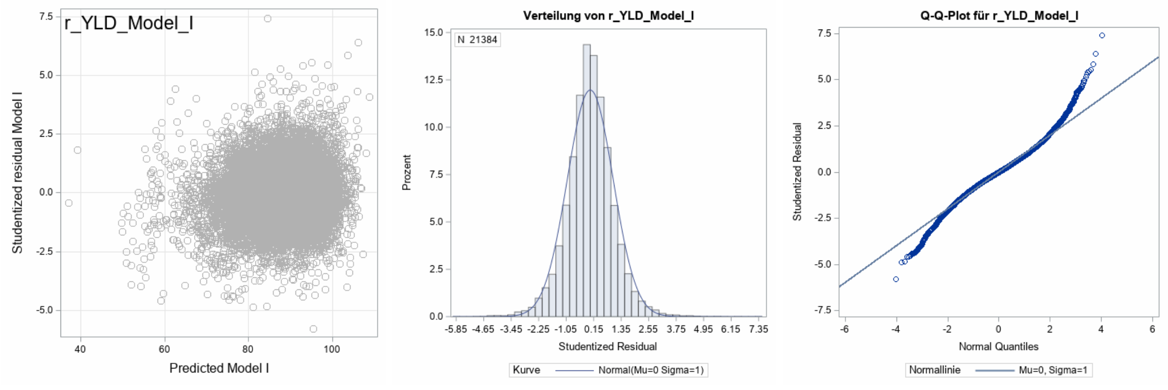

e)

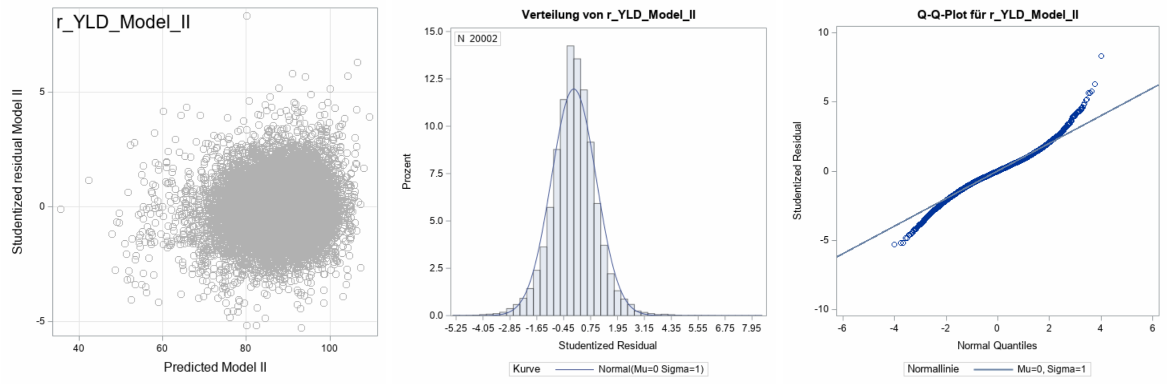

Fig. S1 a) Residual plots for overall trends as modelled by Eqs. (2a), (2c), **b)** Residual plots for variety mean trends as modelled by Eq. (3), **c)** Residuals plots for age trends as modelled by Eqs. (6a), (6b) and (6c), **d)** Residual plots for Model I given by Eq. (7a), **e)** Residual plots for Model II given by Eq. (7b).

YLD Grain yield; *MLD* Mildew; *BNR* Brown rust; *STB* Septoria tritici blotch*;* *SNB* Septoria nodorum blotch; *YLR* Yellow rust; *DTR* Tan spot; *I1* Intensity 1;

References

Bock C. H., Poole G. H., Parker P. E., Gottwald T. R. 2010. Plant Disease Severity Estimated Visually, by Digital Photography and Image Analysis, and by Hyperspectral Imaging. Critical Reviews in Plant Sciences 29:59–107.

Hartung, K., Piepho, H.P. (2007): Are ordinal rating scales better than percent ratings? - A statistical and “psychological” view. Euphytica 155, 15-26.
